# Revised Timeline and Distribution of the Earliest Diverged Human Maternal Lineages in Southern Africa

**DOI:** 10.1371/journal.pone.0121223

**Published:** 2015-03-25

**Authors:** Eva K. F. Chan, Rae-Anne Hardie, Desiree C. Petersen, Karen Beeson, Riana M. S. Bornman, Andrew B. Smith, Vanessa M. Hayes

**Affiliations:** 1 Laboratory for Human Comparative and Prostate Cancer Genomics, Garvan Institute of Medical Research, 384 Victoria Street, Darlinghurst, NSW, 2010, Australia; 2 Faculty of Medicine, University of New South Wales Australia, Randwick, NSW, Australia; 3 J. Craig Venter Institute, 4120 Torrey Pines Road, La Jolla, California, 92037, United States of America; 4 School of Health Systems and Public Health, University of Pretoria, Hatfield, South Africa; 5 Department of Archaeology, University of Cape Town, Rondebosch, South Africa; 6 Central Clinical School, The University of Sydney, Camperdown, NSW, Australia; University of Florence, ITALY

## Abstract

The oldest extant human maternal lineages include mitochondrial haplogroups L0d and L0k found in the southern African click-speaking forager peoples broadly classified as Khoesan. Profiling these early mitochondrial lineages allows for better understanding of modern human evolution. In this study, we profile 77 new early-diverged complete mitochondrial genomes and sub-classify another 105 L0d/L0k individuals from southern Africa. We use this data to refine basal phylogenetic divergence, coalescence times and Khoesan prehistory. Our results confirm L0d as the earliest diverged lineage (∼172 kya, 95%CI: 149–199 kya), followed by L0k (∼159 kya, 95%CI: 136–183 kya) and a new lineage we name L0g (∼94 kya, 95%CI: 72–116 kya). We identify two new L0d1 subclades we name L0d1d and L0d1c4/L0d1e, and estimate L0d2 and L0d1 divergence at ∼93 kya (95%CI:76–112 kya). We concur the earliest emerging L0d1’2 sublineage L0d1b (∼49 kya, 95%CI:37–58 kya) is widely distributed across southern Africa. Concomitantly, we find the most recent sublineage L0d2a (∼17 kya, 95%CI:10–27 kya) to be equally common. While we agree that lineages L0d1c and L0k1a are restricted to contemporary inland Khoesan populations, our observed predominance of L0d2a and L0d1a in non-Khoesan populations suggests a once independent coastal Khoesan prehistory. The distribution of early-diverged human maternal lineages within contemporary southern Africans suggests a rich history of human existence prior to any archaeological evidence of migration into the region. For the first time, we provide a genetic-based evidence for significant modern human evolution in southern Africa at the time of the Last Glacial Maximum at between ∼21–17 kya, coinciding with the emergence of major lineages L0d1a, L0d2b, L0d2d and L0d2a.

## Introduction

Tracing patterns of maternally inherited human mitochondrial genome (mtDNA) variation in contemporary populations has been an invaluable resource for studying anatomically modern human evolution. These studies provided the first genetic evidence for the significant role southern Africa has played in shaping anatomically modern humans [1,2]. Further, complete mtDNA sequencing has dramatically improved the resolution of the global human phylogenetic tree and helped refine estimations of lineage divergence and demographic history. While mounting genetic and phylogeographic evidence places the root of mtDNA phylogeny within southern Africa [2–5], there are also archaeological and oesteological evidence supporting over 100 thousand years of modern human existence in the region [6–9].

The deepest rooting clade of the human mtDNA phylogeny is the L0 macro-haplogroup, estimated to date to between 154 thousand years ago (kya) to 142 kya [2,3,5,10,11]. Within this clade, L0d and L0k are thought to be the earliest extant offshoots of the phylogeny and are largely restricted to the click-speaking forager peoples of southern Africa, the Khoesan [2–4]. Khoesan is a compound word and blanket term covering two groups of peoples: the ‘Khoe’ (Khoekhoe or Khoikhoi) herder-gatherers and the ‘San’ (Saan or Bushmen) hunter-gatherers. Sharing many physical and linguistic characteristics, the Khoe and San are, arguably, culturally distinct with independent prehistories.

Although the exact origins of the southern African Khoesan people are not fully defined, several consensuses have emerged. In brief, Ju-‡Hoan and Tuu speaking San people are thought to be the indigenous hunter-gatherers occupying most of southern Africa prior to the arrival of current day Khoe-Kwadi-speaking Khoe-people [12–15]. Archaeological evidence suggests Khoe people with domesticated caprines migrated from a central African region, traversing the northerly-to-southerly expanse of present day Namibia [16] and reaching the most southwestern tip of the continent by 2 kya [17,18]. This migration is thought to have been driven by a “bow-wave” effect created by the influx of Iron-Age agriculturalist Bantu speakers from west Africa [19]. The Southern Bantu people entered northeastern South Africa roughly 1.5 kya [19], while the Southwestern Bantu entered northern Namibia roughly 400 ya [20,21]. European arrival in the 17^th^ century further displaced many of the existing ethnic groups, as well as contributing to new admixed populations [22].

Although L0d/L0k are the oldest known extant maternal lineages of modern human, these two haplogroups have received little attention until recently [5,23]. These studies have proposed new coalescent times and introduced new subclades (e.g. L0d2d and L0k1a/L0k1b). Because of the vast diversity inherent within the L0-branch of the phylogeny, we assert more extensive sampling and deeper sequencing of mtDNA are required to refine timeline estimates, while continuing to identify new subclades. To address this, we recruited a panel of southern Africans representing the deepest L0 lineages to further mine the mtDNA diversity within this geographic and phylogenetic region. In this study, we generated 77 complete mtDNA genomes and haplogrouped a further 105, which we then merged with previously published datasets of regional and/or haplogroup relevance [5,24–26] to further define basal phylogenetic divergence, coalescence times and demographic history for ancient modern human lineages.

## Materials and Methods

### Study Populations

**Fig 1:**
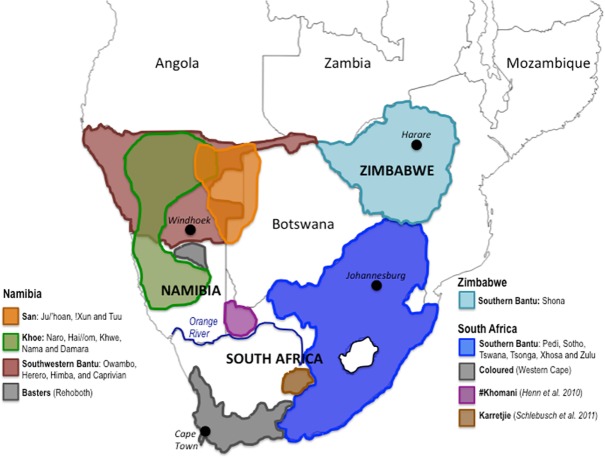
Map of study recruitment areas. Shown is a map of southern Africa depicting United Nations-defined zoned countries. Participants were recruited within the borders of South Africa and Namibia. However, individuals may report place of birth as South Africa, Namibia, Angola, Botswana, or Zimbabwe. Highlighted are geographical distributions and classifications of contemporary populations included in this study. Study participants (n = 182) were defined by place of birth and are broadly classified as *San* (orange) and *Khoe* (green) from Namibia, or Khoesan-ancestral (non-Khoesan with a Khoesan contribution), including the *Basters* (grey) and *Southwestern Bantu* (maroon) from Namibia and the *Coloured* (grey) and *Southern Bantu* (blue) from South Africa. Two Southern Bantu reported Zimbabwe as their place of birth (light blue). Previously published data for the South African #Khomani (purple, n = 32) [25] and Karretjie people (brown, n = 31) [26] has been included and distribution based on reported population densities.

Refer to S1 Table for a summary table of the population groups, including population naming, sample size, and countries of birth; and Fig. 1 for geographical locations of participant recruitment. Additional information provided online at http://garvan.org.au/research/cancer/human-comparative-and-prostate-cancer-genomics/.

***Khoesan*** (n = 67) were recruited from the borders of Namibia and linguistically classified as *San* (n = 26) or *Khoe* (n = 41). Residing in the inland semi-desert regions, the San self-identified as Ju/’hoan (n = 15),! Xun (n = 10), or Tuu-speaker (n = 1). All participants were extensively interviewed regarding their heritage, cultural practices, language, use of population identifiers and relatedness. Predominantly recruited within the western Kalahari region, to the northern pans and south along the west coast of Namibia, the Khoe were classified based on speaking a Kwadi-Khoe language [13,27] and self-identified as Naro (n = 10), Hai//om (n = 8), Khwe (n = 2), Nama (n = 11) or Damara (n = 10). While the Naro and Hai//om, and to a lesser extent the Khwe, were practicing subsistence foraging, the Nama (/Awa-khoin) and Damara (‡Nû-khoin) were largely reliant on westernised subsistence. All participants were interviewed.

***Non-Khoesan*** (n = 115) were recruited within the borders of Namibia or South Africa and subdivided into one of four population groups. The *Southwestern Bantu* (n = 10, including Owambo, Herero, Himba and Caprivian) migrated southwards along the west coast into northern Namibia, while the *Southern Bantu* (n = 40, including Shona, Pedi, Sotho, Tswana, Tsonga, Xhosa and Zulu) migrated along the east coast into South Africa. The arrival of European settlers and slaves to the most southern tip of Africa gave rise to the *South African Coloured* (n = 23) and the *Namibian Baster* (n = 42) populations [22]. Previous mtDNA studies have suggested significant San/Khoe maternal contributions to the Coloured [28–31] and more recently the Baster [31]. All participants completed an interview-based demographic questionnaire including ethno-linguistic identification and parental heritages.

### Published data

For the phylogenetic analysis, we included an additional 526 published mtDNA: one L0d2c genome from a ∼2,330 year old skeleton of Khoesan origin [6], six genomes (five San and one Southern Bantu) from [24], 485 haplogroup relevant genomes as described in [5], all 26 L0a (GenBank accession numbers EF184601-EF184608, Q304897-Q304904, JQ045053, JQ045004, JQ044995, JQ044943, JQ044903, JQ044893, JQ044874, JQ044851, JQ044849, JQ044838) and all 7 L0f (accession numbers AY963585, EF184595-EF184600) genomes from NCBI, as well as the Revised Cambridge Reference Sequence (rCRS; NC_012920) and seven Neanderthal genomes (GenBank accessions NC_011137, KC879692, FM865409, FM865407, FM865408, FM865411, FM865410). For the haplogroup frequency analysis, we included two inland semi-desert South African populations showing significant maternal Khoesan heritage, namely the #Khomani (n = 32) [25], and the Karretjie people (n = 31) [26]. Like the Coloured and Baster populations, the #Khomani and Karretjie predominantly speak Dutch-derived Afrikaans. The #Khomani also speak a Nama or a closely related Khoekhoe language, while very few speak the heritage language N||ng, a language of the Tuu family (Tom Güldemann, personal communication).

### Ethics and research Permits

Study subjects were recruited within the borders of Namibia or South Africa. Written or verbal informed consent was obtained and DNA analysis performed under ethics approvals 43/2010 (University of Pretoria, South Africa), IRB’s 2010–126 and 2010–129 (J. Craig Venter Institute, U.S.A.) and HREC#08244 (University of New South Wales, Australia). Verbal consent was only acquired when literacy was absent and only within Khoesan communities in remote areas of Namibia. All verbal information and consenting was performed by VMH using a mutual language, ‘Afrikaans’, with additional local Khoesan-specific translation, and video recorded as approved and required by the Ministry of Health and Social Services of Namibia. Additional research permits were granted from the Department of Health of the Republic of South Africa.

### Mitochondrial Genome Profiling

**Fig 2:**
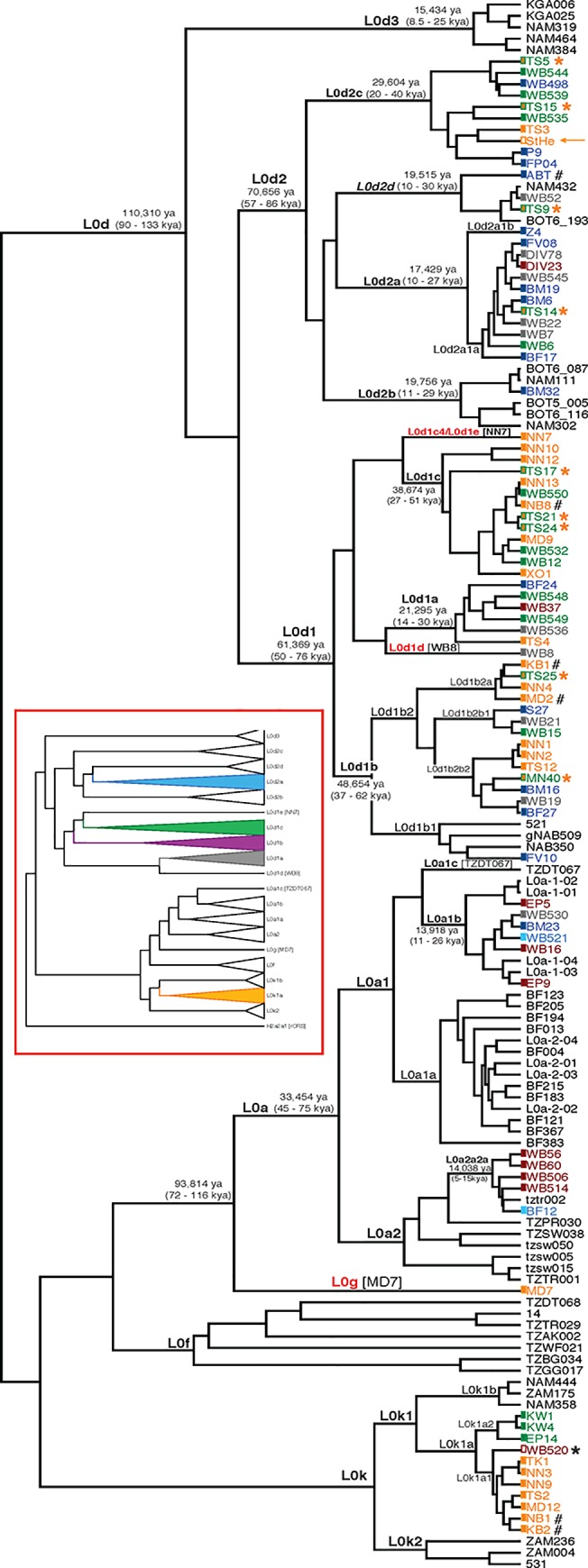
Phylogeny of 139 complete mitochondrial genomes depicting the earliest diverged maternal lineages. The 77 novel southern African mitochondrial genomes sequenced in this study included 32 L0d1, 24 L0d2, 9 L0k1, 1 L0g and 11 L0a. Population representations are colour-coded, by tip labels, as defined in Fig. 1. Co-classifications are indicated by asterisks (*) for peoples defined linguistically as Khoe (green) yet practicing clear forager subsistence, including the Naro, Hai//om and Khwe (orange filled green rectangles). Six previously published mtDNA [24] are indicated by hash marks (#) and one ancient L0d2 (StHe) is indicated by orange arrow [6]. All other publicly obtained mtDNA are shown in black. Mitochondrial haplogroups according to PhyloTree Build 16 [32] are labelled in ‘black’, new haplogroups proposed in previous studies are represented in ‘black italic’, and new haplogroups identified in this study are presented in ‘red’, noting that L0d1e could be L0d1c4. Subclades represented by single mtDNA have sample identifiers provided in square brackets ([]). The simplified tree in the inset (red box) shows the phylogeny inferred from the expanded dataset of 603 genomes; individual genomes are collapsed with each triangle representing the relative diversity of the corresponding haplogroups and subclades. Estiimated coalescent times, including their 95% Highest Probability Density, are shown for the major branches.

Direct amplicon-specific Sanger sequencing (nucleotides 3322 to 4162, numbered according to the rCRS) was used to identify 182 subjects presenting with the L0-haplogroup, as indicated by the C3516A variant but lacking the T4312C variant (indicative of the non-L0 lineage). 77 individuals were randomly selected for whole mtDNA sequencing. In brief, touch-down long-range amplification and the Platinum Taq DNA Polymerase HiFi kit (Invitrogen) was used to generate overlapping amplicons of ∼7.2 Kb and ∼9.7 Kb and purified using AMPure XP beads (Agencourt). PCR products were sheared using the Covaris E220, sequencing libraries constructed using the standard Illumina protocol, sized using AMPure XP beads, and indexes introduced to the adaptor sequences. After purification, the libraries underwent quantification using the High Sensitivity DNA Kit for the Agilent Bioanalyzer, a sampling underwent additional quantification using the KAPA SYBR FAST qPCR Kit (Kapa Biosystems), and data used for creating a single library pool prior to generating 100 bp single-read sequences using a single lane on the Illumina Genome Analyzer IIx. A median of 235,473 matched reads per individual were used to assemble complete mitochondrial genomes using CLC Genomics Workbench version 6.5.1 (http://www.clcbio.com) with default parameters, generating 277-fold to 5,217-fold coverage (S1 Fig.). Haplogroup assignments, and hence phylogenetic clades, were according to PhyloTree Build 16 (www.phylotree.org, [32]; Fig. 2 and S2 Fig. and S2 Table). These 77 mtDNA have been deposited in GenBank with accession numbers KJ669103-KJ669157 and KJ669159-KJ669180. Individuals that did not undergo complete mtDNA sequencing (n = 105) were further haplogrouped using L0d and L0k specific markers and two Sanger sequenced amplicons covering nucleotide positions 3322–4162 and 4344–5995 (S3 Table).

### Phylogenetic Analysis and Age Estimations

Multiple sequence alignment of the 603 complete mtDNA sequences was performed using MUSCLE [33] with default parameters. The Bioconductor package Biostrings [34] was used to partition aligned sequences into four datasets: a focused sample set of 146 genomes and a full sample set of all 603 genomes; and for each, a coding region subset of 15,447 bases (excluding control regions 1–576 and 16024–16569) and a whole genome set of 16,531 bases (excluding two poly-C runs at 303–315 and 16182–16194, a AC run at 515–525, and the mutational hotpot at 16519). The focus set differ from the full sample set in that the former contained only 21 of the 485 mtDNA from [5]: 11 genomes from three haplogroups (5 L0d3, 3 L0k1b, and 3 L0k2) not represented in the current study group, 3 randomly chosen L0d1 genomes to help place FV10 (L0d1b1), 5 random L0d2b genomes to help place BM32 (L0d2b1b), and 2 random L0d2d genomes to confirm the identification of this new haplogroup.

Phylogenies of the expanded mtDNA datasets were estimated with FastTree v2.1.7 [35] using the generalized time-reversible model (-gtr) with four rounds of subtree-prune-regraft moves (-spr 4) and rescaling branch lengths to optimize the gamma20 likelihood (-gamma). Bayesian phylogenetic inferences and divergence times for the focus mtDNA datasets were calculated using BEAST v1.7.5 [36] with 50 million MCMC chains sampling every 5,000 steps and discarding the first 10% as burn-in. Assumptions and priors included: general time reversible (GTR) nucleotide substitution model, discrete gamma distribution (G) with invariant sites (I) for modelling site heterogeneity, and a constant population size coalescent tree prior. Two clock models, a strict clock and an uncorrelated lognormal distributed clock, were examined. Though there was no significant difference between the two models (Bayes Factor <1.0), likelihood values of the relaxed clock model was consistently better, thus this model was used in this paper. Estimates of coalescent times were calculated using two mutation rates: 1.26x10^−8^ substitutions per nucleotide per year for the coding region [11], and 1.67x10^−8^ for the whole genome [10]. Unless stated otherwise, tMRCA (time to most recent common ancestor) derived from whole mtDNA data using the whole genome-specific rate were reported in this paper. Phylogenetic trees were rooted to seven Neanderthal mtDNA and the rCRS reference (haplogroup H2a2a1). Tree visualization and annotation was done using FigTree v1.4.0 (http://tree.bio.ed.ac.uk/software/figtree/). All other analyses were performed using The R Statistical Software [37] and the R/APE package [38].

## Results and Discussion

### Participant classification

Participants were broadly classified as Khoesan or non-Khoesan, with further population specific identification and participant numbers outlined in S1 Table. Participants were recruited from the borders of Namibia or South Africa and their place of birth recorded (Fig. 1). Contemporary Khoesan from Namibia were linguistically classified as ‘*San*’, specifically Ju/’hoan,! Xun or Tuu speakers, or ‘*Khoe*’ (Khoe-Kwadi/Nama-speakers), specifically Naro, Hai//om, Khwe, Nama or Damara. It should be noted that linguistic classifications as used in this study do not always reflect cultural classification. Notably, while the Naro and Hai//om from this study speak a Khoe-Kwadi language (Khoe), they are culturally hunter-gatherers (San) [39]. Non-Khoesan participants were classified as Bantu, specifically ‘*Southern Bantu’* (South Africa) or ‘*Southwestern Bantu’* (Namibia), representing easterly and westerly southward migrations, respectively [17], or South African ‘*Coloured’* or Namibian ‘*Baster’*, the latter populations having arisen as a result of European colonization and slave trade at the then Cape of Good Hope (now Cape Town, South Africa) [18].

### Mitochondrial genome profiling

A total of 182 participants (S1 Table) were identified as carrying the L0-defining C3516A mtDNA haplogroup marker. Of these, 77 were randomly selected for complete mtDNA sequencing (including 18 San, 22 Khoe, 17 Southern Bantu, 10 Southwestern Bantu, and 10 Coloured/Basters) using long-range amplification and Illumina GAIIx sequencing, achieving an average coverage of 1,421-fold with sequencing statistics outlined in S1 Fig. To allow a comprehensive analysis of the earliest diverged maternal modern human lineages, we used a focused dataset of 139 complete mtDNA for the phylogenetic analysis (Fig. 2 and coding region only represented in S2 Fig.), which included the 77 contemporary southern African genomes (this study), an ancient Khoesan St Helena skeleton defined as haplogroup L0d2c [6], six previously reported geographically relevant genomes [24], a subset of 21 haplogroup relevant genomes from a recently published study [5], and 33 publically available genomes belonging to the L0a (n = 26) or L0f (n = 7) haplogroup. An expanded dataset, including the focus set as well as the remaining 464 genomes reported in [5], was assessed further for phylogenetic confirmation (n = 603).

Excluding L0d3, all major Khoesan-containing L0 haplogroups were represented by the 77 complete mitochondrial genomes sequenced within this study, specifically L0d1’2 (n = 56) and L0k1 (n = 9), as well as a newly named L0g haplogroup (n = 1). The remaining 11 genomes represented the early-derived non-Khoesan L0a lineage. The expanded dataset (n = 595) allowed for subclade specific classifications per PhyloTree Build 16 [32] (tabulated in S2 Table). To further elucidate the distribution of the L0d and L0k lineages within contemporary southern African populations, an additional 105 individuals carrying the L0-defining marker and presenting with either the T4232C (L0d, n = 99) or G4541A and G4907C (L0k, n = 6) variants, were further analysed using 11 L0d and three L0k haplogroup specific markers as described in S3 Table. We merged our data (n = 182) with that previously published, adding two additional ethnic groups to our analysis including the #Khomani (n = 32) [25] and Karretjie (n = 31) [26], as well as six extra samples to our existing population groups [24] (total n = 251). We used this data to establish a comprehensive frequency estimation of the early-diverged southern African L0 maternal lineages based on population identity and lineage representation (Table 1).

**Table 1:** Southern African distributions of L0 maternal haplogroups from 251 individuals.

mtDNA Haplogroups	San	Khoe	#Khomani (Henn et al. 2011)	Karretjie (Schlebusch et al. 2011)	Baster (this study)	Coloured (this study)	Southern Bantu (this study)^2^	SWest Bantu (this study)	Total
	(this study)^1^	(this study)							
**L0d2a**	2 (6.5%)	6 (14.6%)	-	18 (58.1%)	14 (33.3%)	8 (34.8%)	17 (41.5%)	1 (10%)	66
*L0d2b*	-	1 (2.4%)	-	-	5 (11.9%)	-	1 (2.4%)	-	7
*L0d2c*	1 (3.2%)	7 (17.1%)	-	-	2 (4.8%)	-	3 (7.3%)	-	13
*L0d2d*	-	2 (4.9%)	-	-	1 (2.4%)	-	1 (2.4%)	-	4
**L0d1a**	1 (3.2%)	4 (9.8%)	14 (43%)	2 (6.5%)	11 (26.2%)	6 (26.1%)	4 (9.8%)	1 (10%)	43
**L0d1b**	8 (25.8%)	7 (17.1%)	16 (50%)	6 (19.4%)	8 (19.1%)	6 (26.1%)	10 (24.4%)	-	61
**L0d1c**	6 (19.4%)	8 (19.5%)	-	-	-	-	-	-	15
*L0d1d (new)*	-	-	-	-	-	1 (4.3%)	-	-	1
*L0d1e (new)*	1 (3.2%)	-	-	-	-	-	-	-	1
*L0d3*	-	1 (2.4%)	-	5 (16.1%)	-	2 (8.7%)	2 (4.9%)	-	10
*L0g (new)*	1 (3.2%)	-	-	-	-	-	-	-	1
**L0k1**	11 (35.5%)	5 (12.2%)	-	-	-	-	-	1 (10%)*	17
L0a	-	-	2 (7%)	-	1 (2.4%)	-	3 (7.3%)	7 (70%)	13
**Total**	**31**	**41**	**32**	**31**	**42**	**23**	**41**	**10**	**251**

Haplogroups in bold were found to occur at high frequencies within southern African populations, those in italic were rare and likely represent largely extinct maternal lineages. New lineages identified in this study are labeled as such. It should be noted that L0a did not receive a thorough assessment in our study and therefore percentages are not true reflections of the overall prevalence.

*The single Southwestern Bantu presenting with an L0k1 maternal lineage was the only unclassified individual in our study who documented himself as Angolan without any further clarification.

^1^The San include 5 previously published in [25].

^2^The Southern Bantu population identifier included a single published mtDNA (ABT) in [25].

### Phylogenetic relationships and coalescent times

Phylogenetic relationships and coalescent times (time to most recent common ancestor, tMRCA) were estimated using the focus dataset with a Bayesian MCMC approach (BEAST v1.7.5; [36]) with a constant population size coalescent model as the tree prior and a relaxed clock model. Coalescent times were estimated using two mutation rates, one specific to the coding region, 1.26x10^−8^ [11], the other to the whole genome, 1.67x10^–8^ [10]. It should be noted that tMRCA estimates using a coding region-specific rate on only the coding region of the mtDNA were highly comparable to those using a whole genome-specific rate on the whole mtDNA (Table 2 and further outlined in S4 Table and S5 Table). In contrast, estimates using a coding region-specific rate with whole mtDNA data were notably inflated likely due to the non-coding regions (i.e. control regions) being more variable than the coding region [10,40]. Similarly, estimates using a whole mtDNA-specific rate on coding region data were deflated for the same reason.

**Table 2:** Estimated coalescence times for the major southern African L0d/L0k mitochondrial genome haplogroups identified.

	Mishmar Rate: 1.26x^−8^		Soares Rate: 1.67x^−8^	
	for coding region		for whole genome	
	Coding Region	Whole Genome	Coding Region	Whole Genome
**Coalescent times**				
L0	**173,117**	224,857	132,769	**172,250**
L0d	**113,342**	144,004	86,741	**110,310**
L0d3	**16,718**	20,412	12,744	**15,434**
L0d1	**57,641**	80,302	44,183	**61,369**
L0d1a	**21,807**	27,954	16,669	**21,295**
L0d1b	**43,730**	63,880	33,376	**48,654**
L0d1c	**33,961**	50,552	26,093	**38,674**
L0d2	**71,576**	92,358	54,770	**70,656**
L0d2a	**18,810**	22,799	14,282	**17,429**
L0d2b	**26,702**	25,727	20,595	**19,756**
L0d2c	**29,508**	38,726	22,517	**29,604**
L0d2d	**24,009**	25,354	18,234	**19,515**
L0k	**45,601**	63,065	34,996	**48,407**
L0k1	**33,428**	44,760	25,670	**34,198**
L0k1a	**15,430**	19,314	11,809	**14,796**
L0k1a1	**10,302**	11,914	7,858	**9,136**
L0k1a2	**7,099**	9,863	5,482	**7,511**
L0a	**57,331**	77,552	43,803	**59,701**
L0a1b	**20,902**	23,645	15,954	**17,857**
L0a2a2a	**10,828**	12,098	8,354	**9,393**
**Divergence times**				
L0g	**91,625**	122,369	70,083	**93,814**
L0d1d	**46,321**	57,930	37,607	**44,084**

Two mutation rates were used: 1.26x10^−8^ mutations per nucleotide per year for the coding region [11] and 1.67x10^−8^ for the whole genome [10]. Estimated were calculated using 15,447 bases of the coding region and 16,531 bases of the whole genome, excluding known hypervariable sites.

Our reconstructed phylogeny concur that L0d is the earliest extant human maternal lineage sharing a MRCA with the L0a’b’f’g’k sister branch ∼172 kya (95%CI: 149–199 kya), followed by L0k, sharing a MRCA with L0a’b’f’g ∼159 kya (95%CI: 136–183 kya). There was an obvious lack of L0f representation in southern Africa, concurring with previous reports [3]. We identify a new, likely Khoesan-specific, maternal lineage L0g, which together with sister clade, L0a, shared a common ancestor with L0f ∼133.8 kya (95%CI: 114–156 kya). Our estimated L0d coalescent time of ∼110 kya (95%CI: 90–133 kya), is in close approximation with [2] (∼101 kya +/- 10 kya) and [5] (∼95 kya, 95%CI:79–121 kya) both of which used only coding region polymorphisms. Also, our data suggests L0d2 emerged ∼70.6 kya, L0d1 ∼61.3 kya and L0d3 ∼15.4 kya. While significant archaeological finds around the southern coast of Africa suggest 100 thousand years of modern human activity [8,41,42], whether these early humans carried L0d maternal lineages, specifically L0d1’2 subclades, remains to be ascertained. Below we present haplogroup specific observations.

### Haplogroup specific dispersals and frequencies

#### Haplogroup L0a

We identified 11 new L0a complete mitochondrial genomes, confirming exclusivity of this lineage to Bantu-speakers. Today, widely distributed throughout eastern, central and southern Africa [2,10,43], L0a2 has specifically been associated with an early southerly Bantu expansion [44,45]. Therefore, we speculate the emergence of L0a1 (∼41.7 kya, 95%CI:30–55 kya) and L0a2 (∼38.2 kya, 95%CI:27–50 kya) occurred outside of southern Africa (i.e. before influx of Bantu people into southern Africa). Within this study two L0a haplogroups predominate. L0a1b (six complete genomes) emerging ∼17.9 kya (95%CI:11–26 kya) is represented by people originating from both the southeasterly (Southern Bantu) and southwesterly (Southwestern Bantu) Bantu expansion events. L0a2a2a (five complete genomes) emerging ∼9.4 kya (95%CI:4.7–15.3 kya) is limited to descendants of the earlier southeasterly migration. It should be noted that, this latter haplogroup was not actively sought after in this study due to previously reported non-Khoesan heritage [46]. Thus, divergence time estimates as well as frequency of the L0a haplogroup within southern African populations may not be accurately reflected here.

#### Haplogroup L0k

Once sharing a common ancestor with L0a’b’f, and likely the new lineage L0g, we concur a Khoesan ancestral heritage for L0k. With nine newly identified complete L0k genomes, we estimate L0k1 and L0k2 split ∼48 kya (95%CI:34–64 kya). We also speculate exclusivity for the subclade L0k1a within the greater Kalahari, specifically 69% San and 31% Khoe. The only non-Khoesan assigned to this haplogroup was an Angolan Southwestern Bantu individual without further ethnolinguistic classification (WB520). We confirmed two new sister clades (L0k1a1/L0k1a2) recently added to PhyloTree Build 16 and estimate their split to have occurred ∼14.8 kya (95%CI:8.5–22.3 kya). In this study, L0k1a1 appears to be San-specific (10/11, 91%) and L0k1a2 Khoe-specific (5/6, 83%). We approximate the tMRCA for San-L0k1a1 to be ∼9.1 kya (95%CI:5–14 kya) and ∼7.5 kya (95%CI:2–14 kya) for Khoe-L0k1a2. Though estimates were based on limited sample sizes, one can speculate a potential correlation between the emergence of a Khoe-specific subclade and the first observation of animal domestication within the region [47].

#### Haplogroup L0d1

Complete L0d1 genomes from this study included all known subgroups, specifically L0d1a (n = 6), L0d1b (n = 13) and L0d1c (n = 11). We estimate tMRCA of L0d1b, L0d1c, and L0d1a at ∼48 kya (95%CI:37–62 kya), ∼39 kya (95%CI:27–51 kya, including NN7, see below), and ∼21 kya (95%CI:14–30 kya), respectively. After L0d2a (see below), L0d1b and L0d1a are the most common L0d haplogroups represented within contemporary southern African populations (24.3% and 17.1%, respectively). While represented within Khoesan (20.8% L0d1b, 6.9% L0d1a), notably elevated frequencies were observed for recently admixed populations, including the #Khomani (50% L0d1b, 44% L0d1a), Baster (19% L0d1b, 26% L0d1a) and Coloured (26% L0d1b, 26% L0d1a). Within L0d1b, we estimate the predominant subclade L0d1b2 to have emerged ∼34 kya (95%CI:24–45 kya), while L0d1b1 emerging ∼27 kya (95%CI:17–38 kya) was represented in this study by a single Southern Bantu genome (FV10, Venda population). Khoesan from this study are predominantly represented within subclades L0d1b2a and L0d1b2b2, while non-Khoesan within subclades L0d1b2b1 and an independent L0d1b2b2 sub-branch. Unlike sister clades L0d1b and L0d1a, L0d1c haplogroup appears to be Khoesan specific, concurring with previous findings [23,48,49].

#### Haplogroup L0d2

The majority of the complete L0d2 genomes from this study belong to L0d2a (12/24, 50%), with a predominance of subclade L0d2a1a (11/12, 91.7%). Haplogroup frequency analysis suggests L0d2a is one of the most, if not the most, common L0d maternal lineage within contemporary southern African populations (66/251, 26.3%) This is in contrast to previous findings suggesting L0d1 to be the most common haplogroup within this region [5,23]. Our data also suggests L0d2a as the most recently dispersed of the L0d1’2 lineages (tMRCA at ∼17 kya, 95%CI:10–27 kya), with a non-Khoesan predominance (58/66, 88%) and lack of San representation (2/31, 6.5%).

Unlike its widely distributed L0d2a sister branch, L0d2b is rare in contemporary populations and likely represents an earlier dispersal (tMRCA at ∼20 kya, 95%CI:11–29 kya). Though predominated by Baster individuals, the expanded phylogenetic dataset suggests a Khoe-Kwadi representation, specifically Hai//om and Gui [5]. Two genomes in this study (represented by a Hai//om and Baster individual), along with the previously sequenced Southern Bantu reference individual ABT [24], form a clear subclade different from L0d2a and L0d2b. Analysis of the expanded dataset confirmed an independent grouping with seven published genomes [5]. The authors had defined this as L0d2d, a new subclade recently recognized in PhyloTree Build 16 and also reported by [23]. Emergence of L0d2d is estimated to have preceded L0d2a, with tMRCA calculated at ∼19.5 kya (95%CI:10–30 kya). Predominated in this study by Khoe speakers (7/13, 54%), L0d2c appears to be the earliest branching L0d2 subclade with tMRCA calculated at ∼29.6 ya (95%CI:20–40 kya), confirming previous findings [23]. Recently, we found this subclade to be present within the first ancient mtDNA generated for the region, specifically from 2,330 year old skeletal remains of a southwest coastal Khoesan individual [6].

### New L0 Haplogroups identified

Three new and likely (linguistically/culturally) extinct independent Khoesan maternal lineages were identified in this study. We name these lineages L0g, L0d1d and L0d1c4/L0d1e (Fig. 2) and although represented by a single genome each, divergence times are speculated.

The L0g genome presented as a L0a sister branch in a! Xun hunter-gatherer (MD7). Carrying all four of the L0a’b’f’k, seven of the nine L0a’b’f and six of the seven stable L0a’b defining variants, only two of the eight known L0a (G11176A and C16188g) and one of the six L0b (T16187C) defining variants were represented. We speculate L0g diverged from L0a ∼93.8 kya (95%CI:71.8–115.7 kya).

Presenting in a Coloured South African (WB8), the new L0d1d genome possessed the L0d1a’c defining mutation C16234T, two of the six L0d1a-defining variants (C152T and T16223C) and only one of the eight L0d1c defining variants (A16129G). Our phylogenetic analysis suggests L0d1d diverged from other L0d1 clades ∼44.1 kya (95%CI:31–58 kya).

Within L0d1c, a single mitochondrial genome from a Ju/’hoan individual (NN7) formed an independent branching within this study and from all known L0d1c genomes (see below). Sharing five of the eight L0d1c defining variants, but not the L0d1a’c variant (C16234T), this genome either represents an independent L0d1 subclade (L0d1e) or an independent L0d1c subclade (L0d1c4). Our phylogenetic analysis suggests L0d1c4/L0d1e diverged from other L0d1 clades ∼38.7 kya (95%CI:27–51 kya).

### Khoesan maternal prehistory

In this study, the term Khoesan has been used to describe participants residing in the boundaries of Namibia and speaking a click-language broadly classified as ‘*San*’ (Ju-‡Hoan/Tuu-speakers) or ‘*Khoe*’ (Khoe-Kwadi/Nama-speakers), while acknowledging the cultural ‘*San*’ (hunter-gatherer) ‘*Khoe*’ (herder-gatherer) distinction. Linguistically and culturally defined as non-Khoesan, the Bantu, Coloured and Baster populations provide an opportunity to speculate on Khoesan prehistory at the southern tip of Africa prior to the arrival of agro-pastoral and European migrants.

Notably absent south of the Orange River (Fig. 1), we concur with others that the L0k1 and L0d1c maternal lineages distinguish contemporary Kalahari Khoesan from non-Khoesan populations [23]. The San identifier in this study is overwhelmingly represented by three haplogroups, specifically L0k1a (35.5%), L0d1b (25.8%) and L0d1c (19.4%), while L0d2 representation is scarce. In contrast, the Khoe identifier has a broad maternal representation, likely reflective of a migratory prehistory. Specifically, L0d1c (19.5%), L0d1b (17.1%), L0d2c (17.1%), L0d2a (14.6%), L0k1a (12.2%) and L0d1a (9.8%). Elevated frequencies of L0d2a within the Southern Bantu (41.5%), Coloured (34.8%) and Baster (33.3%) populations, further supported by previous estimations within the Karretjie (58.1%) [26] and absence within the #Khomani [25], suggests a once broad west to eastern coastal distribution of the L0d2a ancestral lineage. Significant representation of L0d1a within the Coloured and Baster populations (26%), and previously reported for the #Khomani (43%) [25], suggests a south to southwesterly ancestral dispersal. While the L0d1b ancestral lineage appears to have been highly dispersed throughout southern Africa.

We evaluated the clade-specific distribution of the San-dominant haplogroups using an expanded complete mtDNA dataset that included our complete mtDNA and published population-specific completed genomes [5,6,24]. Within L0k1a (n = 84, S3 Fig.), we concur L0k1a1 is isolated to people defined culturally as San (all 52 genomes, excluding the single unclassified Angolan from this study, 100%), while L0k1a2 is biased towards persons culturally classified as Khoe (16/25, 64%). Within L0d1b (n = 137, S4 Fig.), the following novel observations were made: L0d1b1 is largely lacking in persons culturally and linguistically classified as San (2/35, 5.7%), while L0d1b2 predominates within culturally classified San (67/102, 65.7%). San-predominant L0d1b2 subclades include L0d1b2a2, L0d1b2b1a and L0d1b2b2c (only the latter represented in this study). L0d1b1 subclades can be further differentiated into Bantu-specific (L0d1b1b and L0d1b1c) and Khoe-specific (L0d1b1a). A recent study published while our paper was in review identifies two divergent L0d1b1 branches that are restricted to Bantu-speaking populations from southwestern Angola and western Zambia [50]. Unlike L0k1a and L0d1b, the entire L0d1c lineage is dominated by contemporary culturally San populations, with no clear San/Khoe delineation (n = 153, S5 Fig.). The L0d1c genomes from this study are represented within all known lineages, specifically L0d1c1, L0d1c2 and L0d1c3.

## Conclusions

There is general agreement that modern humans originated within Africa and that the most divergent (genetically distinct) populations are found within southern Africa [25,51]. Publication of the first Khoesan genome provided an insight into the extent of this diversity [24] and suggested early divergence of the San 157–108 kya [52]. Sequencing of complete mitochondrial genomes, specifically the L0d and L0k haplogroups, has been invaluable in tracing early divergence of anatomically modern humans within southern Africa [1,44]. In this study, we sequenced 77 complete mitochondrial genomes from contemporary southern African and haplogrouped a further 105. This allowed for the identification of three new haplogroups (L0g, L0d1d and L0d1c4/L0d1e), further refining coalescent times and insights into Khoe/San prehistory.

We estimate that L0d shared a common ancestor with its L0-sister node some 172 kya, the latter including the new Khoesan-specific lineage L0g (L0a’b’f’g’k). We hypothesize L0k shared a common ancestor with L0a’b’f’g roughly 159 kya. We concur that these earliest known derived maternal lineages are common in southern Africa, specifically haplogroups L0d1, L0d2 and L0k1, while L0d3 was rare in this study. We predict L0d2 emerged around 71 kya, followed 10 thousand years later by L0d1. Within L0d1’2, L0d1b emerged around 49 kya and is broadly distributed across both Khoesan and non-Khoesan populations. Unlike L0d1b, L0d1c representation is restricted to contemporary Khoesan with emergence speculated to have occurred roughly 39 kya or 25.5 kya depending on the new subclade classification for a single Ju/’hoan individual (NN7), L0d1c4 or L0d1e, respectively. Like L0d1c, L0k1a representation is restricted to Khoesan peoples inhabiting today the greater inland Kalahari semi-desert region. We speculate L0d2c emerged around 30 kya and although rare, this lineage occurs at increased frequencies within people speaking a Khoe language. Furthermore, we recently identified this lineage within the first ancient mtDNA extracted from a 2,330 year old southwest coastal Khoesan skeleton [6].

Our data points to the Last Glacial Maximum (LGM) as a significant period of human divergence within Southern Africa. Peaking around 21 kya [53], the LGM caused extreme levels of aridity in southern Africa with the highest levels occurring between 19 to 17 kya [54]. Major lineage divergence during this period includes; L0d1a (∼21 kya), L0d2b (∼20 kya), L0d2d (∼20 kya) and L0d2a (∼17 kya). In parallel, southern African fossil records suggest modern Khoesan morphology appeared during this period [55]. This is the first study to provide genetic-based evidence for the potential significant role of the LGM in the emergence, and ultimately the persistence, of the earliest known human maternal lineages within southern Africa.

## Supporting Information

S1 Fig Sequencing statistics for 77 contemporary L0 mtDNA. Method used was long-range amplification, barcoding and pooled Illumina GAIIx sequencing. The median number of reads and matched reads are depicted as box plots with upper and lower ranges. Average length was 100 bases, generating mtDNA coverage ranging from 227-fold to 5,217-fold (average of 1,421-fold).
(PDF)

S2 Fig Phylogeny of 139 complete mitochondrial genomes depicting the earliest diverged maternal lineages, using 15,447 bases of the coding region. The 77 novel southern African mitochondrial genomes sequenced in this study included 32 L0d1, 24 L0d2, 9 L0k1, 1 L0g and 11 L0a. Population representations are colour coded, by tip labels, as defined in Fig. 1, with cultural (fill colour of rectangles) and linguistic (outline colour of rectangles) classifications indicated by rectangles next to tip labels. Co-classifications are indicated by asterisks (*); e.g. Hai//om are co-classified as culturally Bushmen (orange filled rectangles) and linguistically Nama-speakers (green rectangle outline). The previously published Khoesan skeleton assigned to L0d2c [6] is indicated by orange arrow. Six previously published mtDNA [24] are indicated by hash marks (#). All other publicly obtained mtDNA are shown in black. Mitochondrial haplogroups according to PhyloTree Build 16 [32] are labelled in ‘black’, new haplogroups proposed in previous studies are represented in ‘black italic’, and new haplogroups proposed in the current study are presented in ‘red’, noting that L0d1e could be L0d1c4. Subclades represented by single mtDNA have sample identifiers provided in square brackets ([]). The simplified tree in the inset (red box) shows the phylogeny inferred from the expanded dataset of 603 genomes; clades are collapsed with each triangle representing the relative diversity of the corresponding haplogroups and subclades. Estimated coalescent times, including their 95% Highest Probability Density, are shown for the major branches.
(PDF)

S3 Fig Phylogenetic tree for L0k. Phylogeny was inferred using a total of 209 mtDNA, including all 139 mtDNA in the focus set and 70 additional L0k mtDNA from [5]. Haplogroups other than L0k are “collapsed”. Tips of L0k genomes are labelled with the format: Haplogroup (Isolate Name)—Language/Isolate Source [Country], where Isolate Name, Isolate Source, and Country are information included in the corresponding GenBank entries, and Language is the reported spoken language. Tip colours reflect data source: Red = current study, Green = [5], Purple = [24], and Aqua = NCBI.
(PDF)

S4 Fig Phylogenetic tree for L0d1b. Phylogeny was inferred using a total of 258 mtDNA, including all 139 mtDNA in the focus set and 119 additional L0d1b mtDNA from [5]. Haplogroups other than L0d1b are “collapsed”. Tips of L0d1b genomes are labelled with the format: Haplogroup (Isolate Name)—Language/Isolate Source [Country], where Isolate Name, Isolate Source, and Country are information included in the corresponding GenBank entries, and Language is the reported spoken language. Tip colours reflect data source: Red = current study, Green = [5], Purple = [24], and Aqua = NCBI.
(PDF)

S5 Fig Phylogenetic tree for L0d1c. Phylogeny was inferred using a total of 279 mtDNA, including all 139 mtDNA in the focus set and 140 additional L0d1c mtDNA from [5]. Haplogroups other than L0d1c are “collapsed”. Tips of L0d1c genomes are labelled with the format: Haplogroup (Isolate Name)—Language/Isolate Source [Country], where Isolate Name, Isolate Source, and Country are information included in the corresponding GenBank entries, and Language is the reported spoken language. Tip colours reflect data source: Red = current study, Green = [5], Purple = [24], and Aqua = NCBI.
(PDF)

S1 Table Population identifiers used for study participants (n = 182). (PDF)

S2 Table Haplogroups and associated genotypes of mtDNA genomes included in study. Presented are six sub-tables, one for each of haplogroups L0k, L0a, L0g, L0d1, L0d2, and L0d3, showing the alleles of all 595 relevant mtDNA genomes examined in this study, at each defining variant sites of all subclades within the corresponding haplogroup, as indicated in PhyloTree Build 16. Note that data for the seven L0f and rCRS genomes are not included.
(XLSX)

S3 Table Fourteen variants used to identify the major L0d/L0k haplogroups (n = 105). (PDF)

S4 Table Estimated tMRCA for major haplogroups calculated using a coding region-specific mutation rate of 1.26x10^−8^ [11]. (PDF)

S5 Table Estimated tMRCA for major haplogroups calculated using a whole genome-specific mutation rate of 1.67x10^−8^ [10]. (PDF)
