# Supplementary material for: Revised Timeline and Distribution of the Earliest Diverged Human Maternal Lineages in Southern Africa
Source: PLoS One. 2015 Mar 25;10(3):e0121223. doi: 10.1371/journal.pone.0121223 (PMC4373779; doi:10.1371/journal.pone.0121223)
Supplement: S1 Fig — Method used was long-range amplification, barcoding and pooled Illumina GAIIx sequencing. The median number of reads and matched reads are depicted as box plots with upper and lower ranges. Average length was 100 bases, generating mtDNA coverage ranging from 227-fold to 5,217-fold (average of 1,421-fold). (PDF) [file pone.0121223.s001.pdf]

Supporting Information Figure S1

Revised timeline and distribution of the earliest diverged human maternal lineages in southern Africa

Eva K.F. Chan, Rae-Anne Hardie, Desiree C. Petersen, Karen Beeson, Riana M.S. Bornman, Andrew B. Smith and Vanessa M. Hayes

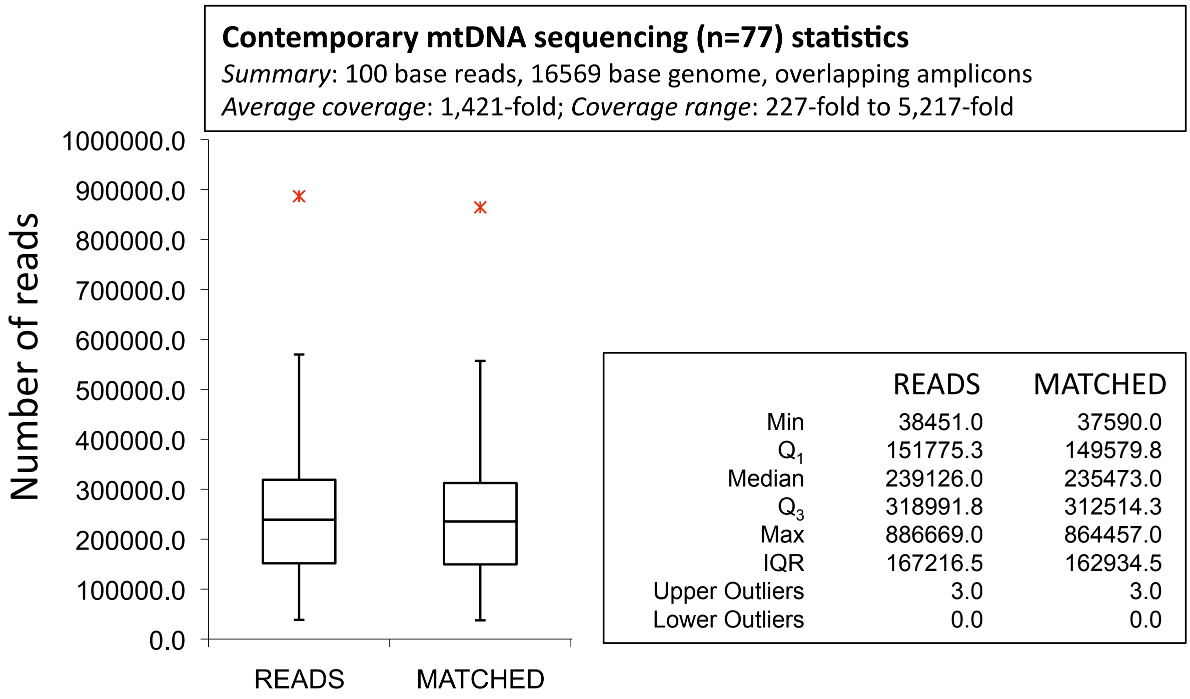

Fig. S1. Sequencing statistics for 77 contemporary L0 mtDNAs. Method used was long-range amplification, barcoding and pooled Illumina GAIIx sequencing. The median number of reads and matched reads are depicted as box plots with upper and lower ranges. Average length was 100 bases, generating mtDNA coverage ranging from 227-fold to 5,217-fold (average of 1,421-fold).
