# Supplementary material for: Revised Timeline and Distribution of the Earliest Diverged Human Maternal Lineages in Southern Africa
Source: PLoS One. 2015 Mar 25;10(3):e0121223. doi: 10.1371/journal.pone.0121223 (PMC4373779; doi:10.1371/journal.pone.0121223)
Supplement: S2 Fig — The 77 novel southern African mitochondrial genomes sequenced in this study included 32 L0d1, 24 L0d2, 9 L0k1, 1 L0g and 11 L0a. Population representations are colour coded, by tip labels, as defined in Fig. 1, with cultural (fill colour of rectangles) and linguistic (outline colour of rectangles) classifications indicated by rectangles next to tip labels. Co-classifications are indicated by asterisks (*); e.g. Hai//om are co-classified as culturally Bushmen (orange filled rectangles) and linguistically Nama-speakers (green rectangle outline). The previously published Khoesan skeleton assigned to L0d2c [6] is indicated by orange arrow. Six previously published mtDNA [24] are indicated by hash marks (#). All other publicly obtained mtDNA are shown in black. Mitochondrial haplogroups according to PhyloTree Build 16 [32] are labelled in ‘black’, new haplogroups proposed in previous studies are represented in ‘black italic’, and new haplogroups proposed in the current study are presented in ‘red’, noting that L0d1e could be L0d1c4. Subclades represented by single mtDNA have sample identifiers provided in square brackets ([]). The simplified tree in the inset (red box) shows the phylogeny inferred from the expanded dataset of 603 genomes; clades are collapsed with each triangle representing the relative diversity of the corresponding haplogroups and subclades. Estimated coalescent times, including their 95% Highest Probability Density, are shown for the major branches. (PDF) [file pone.0121223.s002.pdf]

Supporting Information Figure S2

Revised timeline and distribution of the earliest diverged human maternal lineages in southern Africa

Eva K.F. Chan, Rae-Anne Hardie, Desiree C. Petersen, Karen Beeson, Riana M.S. Bornman, Andrew B. Smith and Vanessa M. Hayes

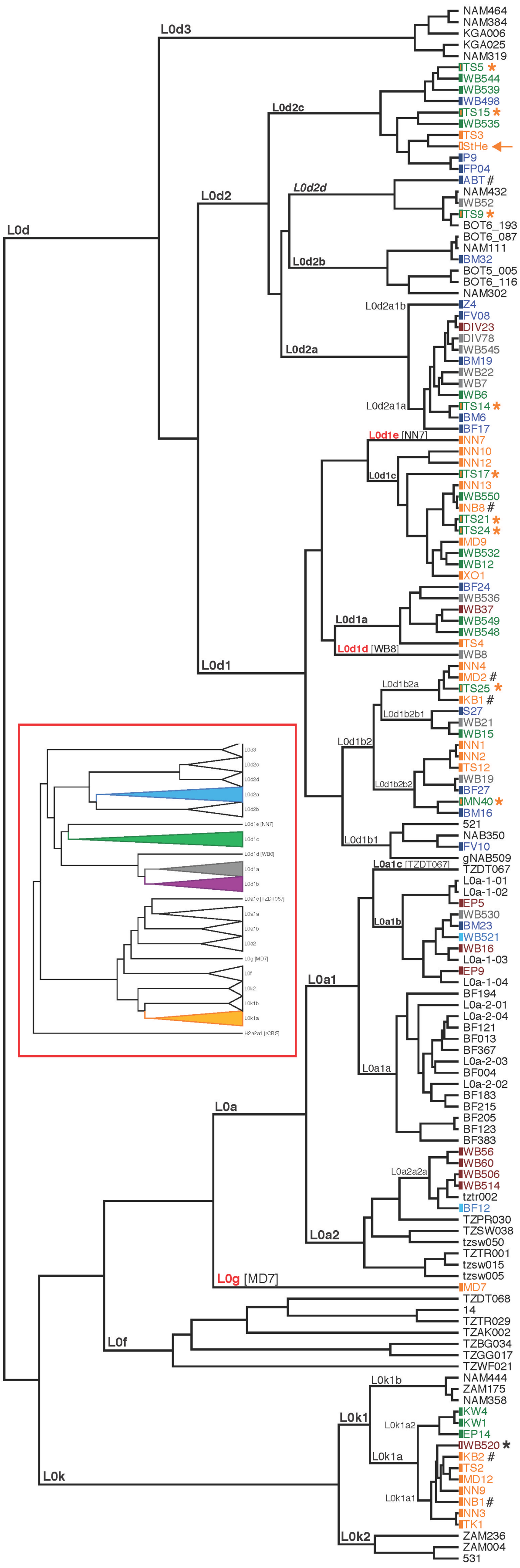

L0d
L0d3
NAM464
NAM384
KGA006
KGA025
NAM319
L0d2
L0d2c
TS5
WB544
WB539
WB498
TS15
WB535
TS3
StHe
P9
FP04
L0d2d
ABT #
NAM432
WB52
TS9
BOT6_193
L0d2b
BOT6_087
NAM111
BM32
BOT5_005
BOT6_116
NAM302
L0d2a
L0d2a1b
Z4
FV08
DIV23
DIV78
WB545
BM19
WB22
WB7
WB6
L0d2a1a
TS14
BM6
BF17
L0d1
L0d1e [NN7]
NN7
L0d1c
NN10
NN12
TS17
NN13
WB550
NB8 #
TS21
TS24
MD9
WB532
WB12
XO1
L0d1a
BF24
WB536
WB37
WB549
WB548
TS4
L0d1d [WB8]
WB8
L0d1b2
L0d1b2a
NN4
MD2 #
TS25
KB1 #
L0d1b2b1
S27
WB21
WB15
L0d1b2b2
NN1
NN2
TS12
WB19
BF27
MN40
BM16
L0d1b1
521
NAB350
FV10
gNAB509
L0a
L0a1
L0a1c [TZDT067]
TZDT067
L0a1b
L0a-1-01
L0a-1-02
EP5
WB530
BM23
WB521
WB16
L0a-1-03
EP9
L0a-1-04
L0a1a
BF194
L0a-2-01
L0a-2-04
BF121
BF013
BF367
L0a-2-03
BF004
L0a-2-02
BF183
BF215
BF205
BF123
BF383
L0a2
L0a2a2a
WB56
WB60
WB506
WB514
tztr002
BF12
TZPR030
TZSW038
tzsw050
TZTR001
tzsw015
tzsw005
L0g [MD7]
MD7
L0f
TZDT068
14
TZTR029
TZAK002
TZBG034
TZGG017
TZWF021
L0k
L0k1
L0k1b
NAM444
ZAM175
NAM358
L0k1a
L0k1a2
KW4
KW1
EP14
WB520
L0k1a1
KB2 #
TS2
MD12
NN9
NB1 #
NN3
TK1
L0k2
ZAM236
ZAM004
531

Fig. S2. Phylogeny of 139 complete mitochondrial genomes depicting the earliest diverged maternal lineages, using 15,447 bases of the coding region. The 77 novel southern African mitochondrial genomes sequenced in this study included 32 L0d1, 24 L0d2, 9 L0k1, 1 L0g and 11 L0a. Population representations are colour coded, by tip labels, as defined in Figure 1, with cultural (fill colour of rectangles) and linguistic (outline colour of rectangles) classifications indicated by rectangles next to tip labels. Co-classifications are indicated by asterisks (*); e.g. Hai//om are co-classified as culturally Bushmen (orange filled rectangles) and linguistically Nama-speakers (green rectangle outline). The previously published Khoesan skeleton assigned to L0d2c (Morris *et al.* 2014) is indicated by orange arrow. Six previously published mtDNA (Schuster *et al.* 2010) are indicated by hash marks (#). All other publicly obtained mtDNA are shown in black. Mitochondrial haplogroups according to PhyloTree Build 16 (Van Oven M and Kayser 2009) are labeled in 'black', new haplogroups proposed in previous studies are represented in 'black italic', and new haplogroups proposed in the current study are presented in 'red', noting that L0d1e could be L0d1c4. Subclades represented by single mtDNA have sample identifiers provided in square brackets ([]). The simplified tree in the inset (red box) shows the phylogeny inferred from the expanded dataset of 603 genomes; clades are collapsed with each triangle representing the relative diversity of the corresponding haplogroups and subclades.
