# Supplementary material for: Revised Timeline and Distribution of the Earliest Diverged Human Maternal Lineages in Southern Africa
Source: PLoS One. 2015 Mar 25;10(3):e0121223. doi: 10.1371/journal.pone.0121223 (PMC4373779; doi:10.1371/journal.pone.0121223)
Supplement: S3 Fig — Phylogeny was inferred using a total of 209 mtDNA, including all 139 mtDNA in the focus set and 70 additional L0k mtDNA from [5]. Haplogroups other than L0k are “collapsed”. Tips of L0k genomes are labelled with the format: Haplogroup (Isolate Name)—Language/Isolate Source [Country], where Isolate Name, Isolate Source, and Country are information included in the corresponding GenBank entries, and Language is the reported spoken language. Tip colours reflect data source: Red = current study, Green = [5], Purple = [24], and Aqua = NCBI. (PDF) [file pone.0121223.s003.pdf]

Supporting Information Figure S3

Revised timeline and distribution of the earliest diverged human maternal lineages in southern Africa

Eva K.F. Chan, Rae-Anne Hardie, Desiree C. Petersen, Karen Beeson, Riana M.S. Bornman, Andrew B. Smith and Vanessa M. Hayes

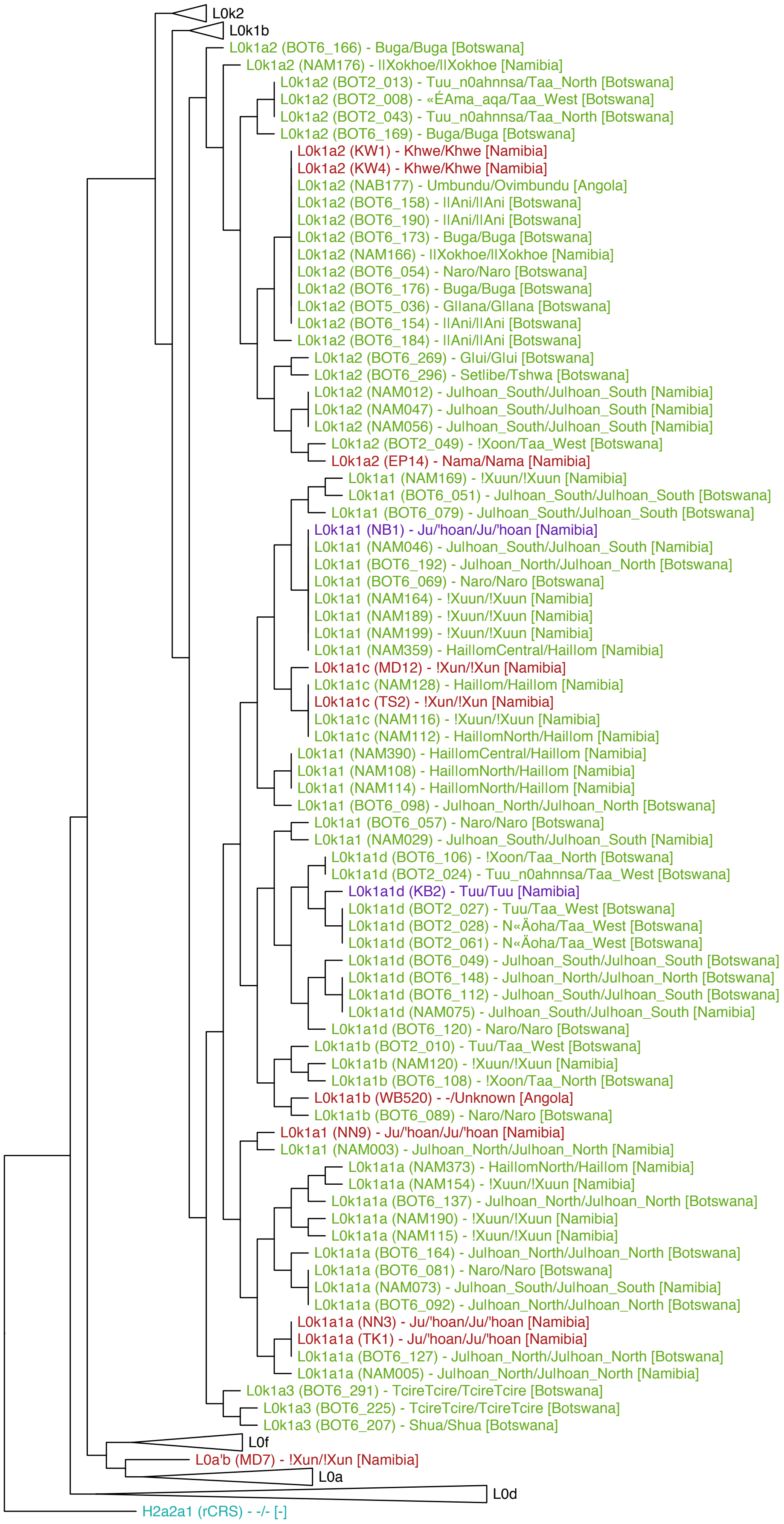
L0k2
L0k1b
L0k1a2 (BOT6_166) - Buga/Buga [Botswana]
L0k1a2 (NAM176) - llXokhoe/llXokhoe [Namibia]
L0k1a2 (BOT2_013) - Tuu_n0ahnnsa/Taa_North [Botswana]
L0k1a2 (BOT2_008) - «ÉAma_aqa/Taa_West [Botswana]
L0k1a2 (BOT2_043) - Tuu_n0ahnnsa/Taa_North [Botswana]
L0k1a2 (BOT6_169) - Buga/Buga [Botswana]
L0k1a2 (KW1) - Khwe/Khwe [Namibia]
L0k1a2 (KW4) - Khwe/Khwe [Namibia]
L0k1a2 (NAB177) - Umbundu/Ovimbundu [Angola]
L0k1a2 (BOT6_158) - llAni/llAni [Botswana]
L0k1a2 (BOT6_190) - llAni/llAni [Botswana]
L0k1a2 (BOT6_173) - Buga/Buga [Botswana]
L0k1a2 (NAM166) - llXokhoe/llXokhoe [Namibia]
L0k1a2 (BOT6_054) - Naro/Naro [Botswana]
L0k1a2 (BOT6_176) - Buga/Buga [Botswana]
L0k1a2 (BOT5_036) - Gllana/Gllana [Botswana]
L0k1a2 (BOT6_154) - llAni/llAni [Botswana]
L0k1a2 (BOT6_184) - llAni/llAni [Botswana]
L0k1a2 (BOT6_269) - Glui/Glui [Botswana]
L0k1a2 (BOT6_296) - Setlibe/Tshwa [Botswana]
L0k1a2 (NAM012) - Julhoan_South/Julhoan_South [Namibia]
L0k1a2 (NAM047) - Julhoan_South/Julhoan_South [Namibia]
L0k1a2 (NAM056) - Julhoan_South/Julhoan_South [Namibia]
L0k1a2 (BOT2_049) - !Xoon/Taa_West [Botswana]
L0k1a2 (EP14) - Nama/Nama [Namibia]
L0k1a1 (NAM169) - !Xuun/!Xuun [Namibia]
L0k1a1 (BOT6_051) - Julhoan_South/Julhoan_South [Botswana]
L0k1a1 (BOT6_079) - Julhoan_South/Julhoan_South [Botswana]
L0k1a1 (NB1) - Ju/'hoan/Ju/'hoan [Namibia]
L0k1a1 (NAM046) - Julhoan_South/Julhoan_South [Namibia]
L0k1a1 (BOT6_192) - Julhoan_North/Julhoan_North [Botswana]
L0k1a1 (BOT6_069) - Naro/Naro [Botswana]
L0k1a1 (NAM164) - !Xuun/!Xuun [Namibia]
L0k1a1 (NAM189) - !Xuun/!Xuun [Namibia]
L0k1a1 (NAM199) - !Xuun/!Xuun [Namibia]
L0k1a1 (NAM359) - HaillomCentral/Haillom [Namibia]
L0k1a1c (MD12) - !Xun/!Xun [Namibia]
L0k1a1c (NAM128) - Haillom/Haillom [Namibia]
L0k1a1c (TS2) - !Xun/!Xun [Namibia]
L0k1a1c (NAM116) - !Xuun/!Xuun [Namibia]
L0k1a1c (NAM112) - HaillomNorth/Haillom [Namibia]
L0k1a1 (NAM390) - HaillomCentral/Haillom [Namibia]
L0k1a1 (NAM108) - HaillomNorth/Haillom [Namibia]
L0k1a1 (NAM114) - HaillomNorth/Haillom [Namibia]
L0k1a1 (BOT6_098) - Julhoan_North/Julhoan_North [Botswana]
L0k1a1 (BOT6_057) - Naro/Naro [Botswana]
L0k1a1 (NAM029) - Julhoan_South/Julhoan_South [Namibia]
L0k1a1d (BOT6_106) - !Xoon/Taa_North [Botswana]
L0k1a1d (BOT2_024) - Tuu_n0ahnnsa/Taa_West [Botswana]
L0k1a1d (KB2) - Tuu/Tuu [Namibia]
L0k1a1d (BOT2_027) - Tuu/Taa_West [Botswana]
L0k1a1d (BOT2_028) - N«Äoha/Taa_West [Botswana]
L0k1a1d (BOT2_061) - N«Äoha/Taa_West [Botswana]
L0k1a1d (BOT6_049) - Julhoan_South/Julhoan_South [Botswana]
L0k1a1d (BOT6_148) - Julhoan_North/Julhoan_North [Botswana]
L0k1a1d (BOT6_112) - Julhoan_South/Julhoan_South [Botswana]
L0k1a1d (NAM075) - Julhoan_South/Julhoan_South [Namibia]
L0k1a1d (BOT6_120) - Naro/Naro [Botswana]
L0k1a1b (BOT2_010) - Tuu/Taa_West [Botswana]
L0k1a1b (NAM120) - !Xuun/!Xuun [Namibia]
L0k1a1b (BOT6_108) - !Xoon/Taa_North [Botswana]
L0k1a1b (WB520) - -/Unknown [Angola]
L0k1a1b (BOT6_089) - Naro/Naro [Botswana]
L0k1a1 (NN9) - Ju/'hoan/Ju/'hoan [Namibia]
L0k1a1 (NAM003) - Julhoan_North/Julhoan_North [Namibia]
L0k1a1a (NAM373) - HaillomNorth/Haillom [Namibia]
L0k1a1a (NAM154) - !Xuun/!Xuun [Namibia]
L0k1a1a (BOT6_137) - Julhoan_North/Julhoan_North [Botswana]
L0k1a1a (NAM190) - !Xuun/!Xuun [Namibia]
L0k1a1a (NAM115) - !Xuun/!Xuun [Namibia]
L0k1a1a (BOT6_164) - Julhoan_North/Julhoan_North [Botswana]
L0k1a1a (BOT6_081) - Naro/Naro [Botswana]
L0k1a1a (NAM073) - Julhoan_South/Julhoan_South [Namibia]
L0k1a1a (BOT6_092) - Julhoan_North/Julhoan_North [Botswana]
L0k1a1a (NN3) - Ju/'hoan/Ju/'hoan [Namibia]
L0k1a1a (TK1) - Ju/'hoan/Ju/'hoan [Namibia]
L0k1a1a (BOT6_127) - Julhoan_North/Julhoan_North [Botswana]
L0k1a1a (NAM005) - Julhoan_North/Julhoan_North [Namibia]
L0k1a3 (BOT6_291) - TcireTcire/TcireTcire [Botswana]
L0k1a3 (BOT6_225) - TcireTcire/TcireTcire [Botswana]
L0k1a3 (BOT6_207) - Shua/Shua [Botswana]
L0f
L0a'b (MD7) - !Xun/!Xun [Namibia]
L0a
L0d
H2a2a1 (rCRS) - -/- [-]

Fig. S3. Phylogenetic tree for L0k. Phylogeny was inferred using a total of 209 mtDNA, including all 139 mtDNA in the focus set and 70 additional L0k mtDNA from Barbieri *et al*. 2013. Haplogroups other than L0k are "collapsed". Tips of L0k genomes are labeled with the format: Haplogroup (Isolate Name) - Language/Isolate Source [Country], where Isolate Name, Isolate Source, and Country are information included in the corresponding GenBank entries, and Language is the reported spoken language. Tip colours reflect data source: Red = current study, Green = Barbieri *et al*. 2013, Purple = Schuster *et al*. 2010, and Aqua = NCBI.
