# Supplementary material for: Revised Timeline and Distribution of the Earliest Diverged Human Maternal Lineages in Southern Africa
Source: PLoS One. 2015 Mar 25;10(3):e0121223. doi: 10.1371/journal.pone.0121223 (PMC4373779; doi:10.1371/journal.pone.0121223)
Supplement: S4 Fig — Phylogeny was inferred using a total of 258 mtDNA, including all 139 mtDNA in the focus set and 119 additional L0d1b mtDNA from [5]. Haplogroups other than L0d1b are “collapsed”. Tips of L0d1b genomes are labelled with the format: Haplogroup (Isolate Name)—Language/Isolate Source [Country], where Isolate Name, Isolate Source, and Country are information included in the corresponding GenBank entries, and Language is the reported spoken language. Tip colours reflect data source: Red = current study, Green = [5], Purple = [24], and Aqua = NCBI. (PDF) [file pone.0121223.s004.pdf]

Supporting Information Figure S4

Revised timeline and distribution of the earliest diverged human maternal lineages in southern Africa

Eva K.F. Chan, Rae-Anne Hardie, Desiree C. Petersen, Karen Beeson, Riana M.S. Bornman, Andrew B. Smith and Vanessa M. Hayes

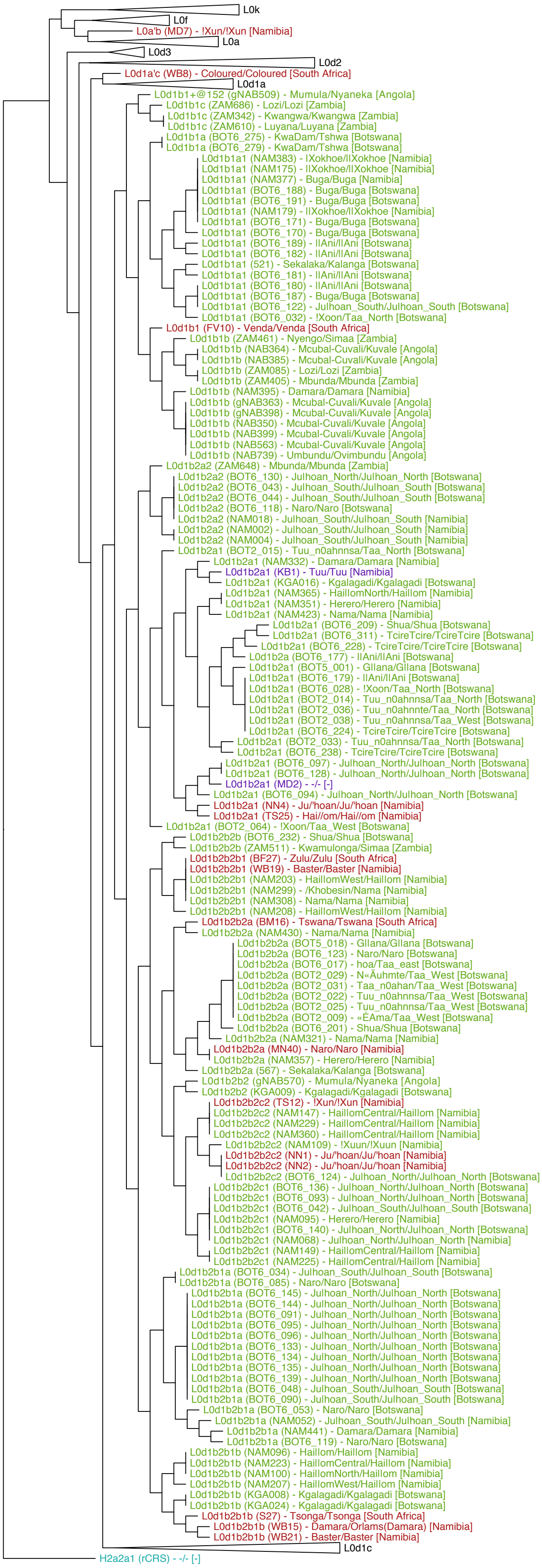
L0k
L0f
L0a'b (MD7) - !Xun/!Xun [Namibia]
L0a
L0d3
L0d2
L0d1a'c (WB8) - Coloured/Coloured [South Africa]
L0d1a
L0d1b1+@152 (gNAB509) - Mumula/Nyaneka [Angola]
L0d1b1c (ZAM686) - Lozi/Lozi [Zambia]
L0d1b1c (ZAM342) - Kwangwa/Kwangwa [Zambia]
L0d1b1c (ZAM610) - Luyana/Luyana [Zambia]
L0d1b1a (BOT6_275) - KwaDam/Tshwa [Botswana]
L0d1b1a (BOT6_279) - KwaDam/Tshwa [Botswana]
L0d1b1a1 (NAM383) - llXokhoe/llXokhoe [Namibia]
L0d1b1a1 (NAM175) - llXokhoe/llXokhoe [Namibia]
L0d1b1a1 (NAM377) - Buga/Buga [Namibia]
L0d1b1a1 (BOT6_188) - Buga/Buga [Botswana]
L0d1b1a1 (BOT6_191) - Buga/Buga [Botswana]
L0d1b1a1 (NAM179) - llXokhoe/llXokhoe [Namibia]
L0d1b1a1 (BOT6_171) - Buga/Buga [Botswana]
L0d1b1a1 (BOT6_170) - Buga/Buga [Botswana]
L0d1b1a1 (BOT6_189) - llAni/llAni [Botswana]
L0d1b1a1 (BOT6_182) - llAni/llAni [Botswana]
L0d1b1a1 (521) - Sekalaka/Kalanga [Botswana]
L0d1b1a1 (BOT6_181) - llAni/llAni [Botswana]
L0d1b1a1 (BOT6_180) - llAni/llAni [Botswana]
L0d1b1a1 (BOT6_187) - Buga/Buga [Botswana]
L0d1b1a1 (BOT6_122) - Julhoan_South/Julhoan_South [Botswana]
L0d1b1a1 (BOT6_032) - !Xoon/Taa_North [Botswana]
L0d1b1 (FV10) - Venda/Venda [South Africa]
L0d1b1b (ZAM461) - Nyengo/Simaa [Zambia]
L0d1b1b (NAB364) - Mcubal-Cuvali/Kuvale [Angola]
L0d1b1b (NAB385) - Mcubal-Cuvali/Kuvale [Angola]
L0d1b1b (ZAM085) - Lozi/Lozi [Zambia]
L0d1b1b (ZAM405) - Mbunda/Mbunda [Zambia]
L0d1b1b (NAM395) - Damara/Damara [Namibia]
L0d1b1b (gNAB363) - Mcubal-Cuvali/Kuvale [Angola]
L0d1b1b (gNAB398) - Mcubal-Cuvali/Kuvale [Angola]
L0d1b1b (NAB350) - Mcubal-Cuvali/Kuvale [Angola]
L0d1b1b (NAB399) - Mcubal-Cuvali/Kuvale [Angola]
L0d1b1b (NAB563) - Mcubal-Cuvali/Kuvale [Angola]
L0d1b1b (NAB739) - Umbundu/Ovimbundu [Angola]
L0d1b2a2 (ZAM648) - Mbunda/Mbunda [Zambia]
L0d1b2a2 (BOT6_130) - Julhoan_North/Julhoan_North [Botswana]
L0d1b2a2 (BOT6_043) - Julhoan_South/Julhoan_South [Botswana]
L0d1b2a2 (BOT6_044) - Julhoan_South/Julhoan_South [Botswana]
L0d1b2a2 (BOT6_118) - Naro/Naro [Botswana]
L0d1b2a2 (NAM018) - Julhoan_South/Julhoan_South [Namibia]
L0d1b2a2 (NAM002) - Julhoan_South/Julhoan_South [Namibia]
L0d1b2a2 (NAM004) - Julhoan_South/Julhoan_South [Namibia]
L0d1b2a1 (BOT2_015) - Tuu_n0ahnnsa/Taa_North [Botswana]
L0d1b2a1 (NAM332) - Damara/Damara [Namibia]
L0d1b2a1 (KB1) - Tuu/Tuu [Namibia]
L0d1b2a1 (KGA016) - Kgalagadi/Kgalagadi [Botswana]
L0d1b2a1 (NAM365) - HaillomNorth/Haillom [Namibia]
L0d1b2a1 (NAM351) - Herero/Herero [Namibia]
L0d1b2a1 (NAM423) - Nama/Nama [Namibia]
L0d1b2a1 (BOT6_209) - Shua/Shua [Botswana]
L0d1b2a1 (BOT6_311) - TcireTcire/TcireTcire [Botswana]
L0d1b2a1 (BOT6_228) - TcireTcire/TcireTcire [Botswana]
L0d1b2a (BOT6_177) - llAni/llAni [Botswana]
L0d1b2a1 (BOT5_001) - Gllana/Gllana [Botswana]
L0d1b2a1 (BOT6_179) - llAni/llAni [Botswana]
L0d1b2a1 (BOT6_028) - !Xoon/Taa_North [Botswana]
L0d1b2a1 (BOT2_014) - Tuu_n0ahnnsa/Taa_North [Botswana]
L0d1b2a1 (BOT2_036) - Tuu_n0ahnnte/Taa_North [Botswana]
L0d1b2a1 (BOT2_038) - Tuu_n0ahnnsa/Taa_West [Botswana]
L0d1b2a1 (BOT6_224) - TcireTcire/TcireTcire [Botswana]
L0d1b2a1 (BOT2_033) - Tuu_n0ahnnsa/Taa_North [Botswana]
L0d1b2a1 (BOT6_238) - TcireTcire/TcireTcire [Botswana]
L0d1b2a1 (BOT6_097) - Julhoan_North/Julhoan_North [Botswana]
L0d1b2a1 (BOT6_128) - Julhoan_North/Julhoan_North [Botswana]
L0d1b2a1 (MD2) - -/- [-]
L0d1b2a1 (BOT6_094) - Julhoan_North/Julhoan_North [Botswana]
L0d1b2a1 (NN4) - Ju/'hoan/Ju/'hoan [Namibia]
L0d1b2a1 (TS25) - Hai//om/Hai//om [Namibia]
L0d1b2a1 (BOT2_064) - !Xoon/Taa_West [Botswana]
L0d1b2b2b (BOT6_232) - Shua/Shua [Botswana]
L0d1b2b2b (ZAM511) - Kwamulonga/Simaa [Zambia]
L0d1b2b2b1 (BF27) - Zulu/Zulu [South Africa]
L0d1b2b2b1 (WB19) - Baster/Baster [Namibia]
L0d1b2b2b1 (NAM203) - HaillomWest/Haillom [Namibia]
L0d1b2b2b1 (NAM299) - /Khobesin/Nama [Namibia]
L0d1b2b2b1 (NAM308) - Nama/Nama [Namibia]
L0d1b2b2b1 (NAM208) - HaillomWest/Haillom [Namibia]
L0d1b2b2a (BM16) - Tswana/Tswana [South Africa]
L0d1b2b2a (NAM430) - Nama/Nama [Namibia]
L0d1b2b2a (BOT5_018) - Gllana/Gllana [Botswana]
L0d1b2b2a (BOT6_123) - Naro/Naro [Botswana]
L0d1b2b2a (BOT6_017) - hoa/Taa_east [Botswana]
L0d1b2b2a (BOT2_029) - N«Äuhmte/Taa_West [Botswana]
L0d1b2b2a (BOT2_031) - Taa_n0ahan/Taa_West [Botswana]
L0d1b2b2a (BOT2_022) - Tuu_n0ahnnsa/Taa_West [Botswana]
L0d1b2b2a (BOT2_025) - Tuu_n0ahnnsa/Taa_West [Botswana]
L0d1b2b2a (BOT2_009) - «EAma/Taa_West [Botswana]
L0d1b2b2a (BOT6_201) - Shua/Shua [Botswana]
L0d1b2b2a (NAM321) - Nama/Nama [Namibia]
L0d1b2b2a (MN40) - Naro/Naro [Namibia]
L0d1b2b2a (NAM357) - Herero/Herero [Namibia]
L0d1b2b2a (567) - Sekalaka/Kalanga [Botswana]
L0d1b2b2 (gNAB570) - Mumula/Nyaneka [Angola]
L0d1b2b2 (KGA009) - Kgalagadi/Kgalagadi [Botswana]
L0d1b2b2c2 (TS12) - !Xun/!Xun [Namibia]
L0d1b2b2c2 (NAM147) - HaillomCentral/Haillom [Namibia]
L0d1b2b2c2 (NAM229) - HaillomCentral/Haillom [Namibia]
L0d1b2b2c2 (NAM360) - HaillomCentral/Haillom [Namibia]
L0d1b2b2c2 (NAM109) - !Xuun/!Xuun [Namibia]
L0d1b2b2c2 (NN1) - Ju/'hoan/Ju/'hoan [Namibia]
L0d1b2b2c2 (NN2) - Ju/'hoan/Ju/'hoan [Namibia]
L0d1b2b2c2 (BOT6_124) - Julhoan_North/Julhoan_North [Botswana]
L0d1b2b2c1 (BOT6_136) - Julhoan_North/Julhoan_North [Botswana]
L0d1b2b2c1 (BOT6_093) - Julhoan_North/Julhoan_North [Botswana]
L0d1b2b2c1 (BOT6_042) - Julhoan_South/Julhoan_South [Botswana]
L0d1b2b2c1 (NAM095) - Herero/Herero [Namibia]
L0d1b2b2c1 (BOT6_140) - Julhoan_North/Julhoan_North [Botswana]
L0d1b2b2c1 (NAM068) - Julhoan_North/Julhoan_North [Namibia]
L0d1b2b2c1 (NAM149) - HaillomCentral/Haillom [Namibia]
L0d1b2b2c1 (NAM225) - HaillomCentral/Haillom [Namibia]
L0d1b2b1a (BOT6_034) - Julhoan_South/Julhoan_South [Botswana]
L0d1b2b1a (BOT6_085) - Naro/Naro [Botswana]
L0d1b2b1a (BOT6_145) - Julhoan_North/Julhoan_North [Botswana]
L0d1b2b1a (BOT6_144) - Julhoan_North/Julhoan_North [Botswana]
L0d1b2b1a (BOT6_091) - Julhoan_North/Julhoan_North [Botswana]
L0d1b2b1a (BOT6_095) - Julhoan_North/Julhoan_North [Botswana]
L0d1b2b1a (BOT6_096) - Julhoan_North/Julhoan_North [Botswana]
L0d1b2b1a (BOT6_133) - Julhoan_North/Julhoan_North [Botswana]
L0d1b2b1a (BOT6_134) - Julhoan_North/Julhoan_North [Botswana]
L0d1b2b1a (BOT6_135) - Julhoan_North/Julhoan_North [Botswana]
L0d1b2b1a (BOT6_139) - Julhoan_North/Julhoan_North [Botswana]
L0d1b2b1a (BOT6_048) - Julhoan_South/Julhoan_South [Botswana]
L0d1b2b1a (BOT6_090) - Julhoan_South/Julhoan_South [Botswana]
L0d1b2b1a (BOT6_053) - Naro/Naro [Botswana]
L0d1b2b1a (NAM052) - Julhoan_South/Julhoan_South [Namibia]
L0d1b2b1a (NAM441) - Damara/Damara [Namibia]
L0d1b2b1a (BOT6_119) - Naro/Naro [Botswana]
L0d1b2b1b (NAM096) - Haillom/Haillom [Namibia]
L0d1b2b1b (NAM223) - HaillomCentral/Haillom [Namibia]
L0d1b2b1b (NAM100) - HaillomNorth/Haillom [Namibia]
L0d1b2b1b (NAM207) - HaillomWest/Haillom [Namibia]
L0d1b2b1b (KGA008) - Kgalagadi/Kgalagadi [Botswana]
L0d1b2b1b (KGA024) - Kgalagadi/Kgalagadi [Botswana]
L0d1b2b1b (S27) - Tsonga/Tsonga [South Africa]
L0d1b2b1b (WB15) - Damara/Orlams(Damara) [Namibia]
L0d1b2b1b (WB21) - Baster/Baster [Namibia]
L0d1c
H2a2a1 (rCRS) - -/- [-]

Fig. S4. Phylogenetic tree for L0d1b. Phylogeny was inferred using a total of 258 mtDNA, including all 139 mtDNA in the focus set and 119 additional L0d1b mtDNA from Barbieri *et al*. 2013. Haplogroups other than L0d1b are “collapsed”. Tips of L0d1b genomes are labeled with the format: Haplogroup (Isolate Name) - Language/Isolate Source [Country], where Isolate Name, Isolate Source, and Country are information included in the corresponding GenBank entries, and Language is the reported spoken language. Tip colours reflect data source: Red = current study, Green = Barbieri *et al*. 2013, Purple = Schuster *et al*. 2010, and Aqua = NCBI.
