# Supplementary material for: Revised Timeline and Distribution of the Earliest Diverged Human Maternal Lineages in Southern Africa
Source: PLoS One. 2015 Mar 25;10(3):e0121223. doi: 10.1371/journal.pone.0121223 (PMC4373779; doi:10.1371/journal.pone.0121223)
Supplement: S5 Fig — Phylogeny was inferred using a total of 279 mtDNA, including all 139 mtDNA in the focus set and 140 additional L0d1c mtDNA from [5]. Haplogroups other than L0d1c are “collapsed”. Tips of L0d1c genomes are labelled with the format: Haplogroup (Isolate Name)—Language/Isolate Source [Country], where Isolate Name, Isolate Source, and Country are information included in the corresponding GenBank entries, and Language is the reported spoken language. Tip colours reflect data source: Red = current study, Green = [5], Purple = [24], and Aqua = NCBI. (PDF) [file pone.0121223.s005.pdf]

Supporting Information Figure S5

Revised timeline and distribution of the earliest diverged human maternal lineages in southern Africa

Eva K.F. Chan, Rae-Anne Hardie, Desiree C. Petersen, Karen Beeson, Riana M.S. Bornman, Andrew B. Smith and Vanessa M. Hayes

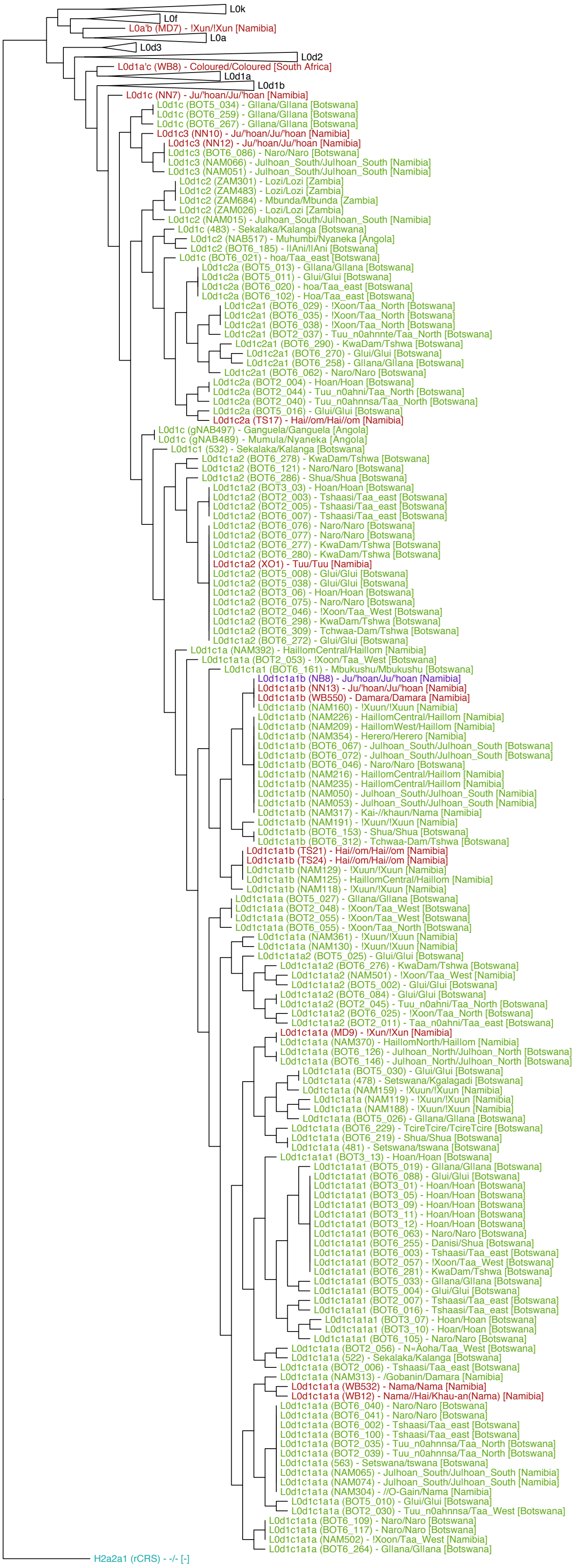

L0k
L0f
L0a'b (MD7) - !Xun/!Xun [Namibia]
L0a
L0d3
L0d2
L0d1a'c (WB8) - Coloured/Coloured [South Africa]
L0d1a
L0d1b
L0d1c (NN7) - Ju/'hoan/Ju/'hoan [Namibia]
L0d1c (BOT5_034) - Gllana/Gllana [Botswana]
L0d1c (BOT6_259) - Gllana/Gllana [Botswana]
L0d1c (BOT6_267) - Gllana/Gllana [Botswana]
L0d1c3 (NN10) - Ju/'hoan/Ju/'hoan [Namibia]
L0d1c3 (NN12) - Ju/'hoan/Ju/'hoan [Namibia]
L0d1c3 (BOT6_086) - Naro/Naro [Botswana]
L0d1c3 (NAM066) - Julhoan_South/Julhoan_South [Namibia]
L0d1c3 (NAM051) - Julhoan_South/Julhoan_South [Namibia]
L0d1c2 (ZAM301) - Lozi/Lozi [Zambia]
L0d1c2 (ZAM483) - Lozi/Lozi [Zambia]
L0d1c2 (ZAM684) - Mbunda/Mbunda [Zambia]
L0d1c2 (ZAM026) - Lozi/Lozi [Zambia]
L0d1c2 (NAM015) - Julhoan_South/Julhoan_South [Namibia]
L0d1c (483) - Sekalaka/Kalanga [Botswana]
L0d1c2 (NAB517) - Muhumbi/Nyaneka [Angola]
L0d1c2 (BOT6_185) - llAni/llAni [Botswana]
L0d1c (BOT6_021) - hoa/Taa_east [Botswana]
L0d1c2a (BOT5_013) - Gllana/Gllana [Botswana]
L0d1c2a (BOT5_011) - Glui/Glui [Botswana]
L0d1c2a (BOT6_020) - hoa/Taa_east [Botswana]
L0d1c2a (BOT6_102) - Hoa/Taa_east [Botswana]
L0d1c2a1 (BOT6_029) - !Xoon/Taa_North [Botswana]
L0d1c2a1 (BOT6_035) - !Xoon/Taa_North [Botswana]
L0d1c2a1 (BOT6_038) - !Xoon/Taa_North [Botswana]
L0d1c2a1 (BOT2_037) - Tuu_n0ahnnte/Taa_North [Botswana]
L0d1c2a1 (BOT6_290) - KwaDam/Tshwa [Botswana]
L0d1c2a1 (BOT6_270) - Glui/Glui [Botswana]
L0d1c2a1 (BOT6_258) - Gllana/Gllana [Botswana]
L0d1c2a1 (BOT6_062) - Naro/Naro [Botswana]
L0d1c2a (BOT2_004) - Hoan/Hoan [Botswana]
L0d1c2a (BOT2_044) - Tuu_n0ahni/Taa_North [Botswana]
L0d1c2a (BOT2_040) - Tuu_n0ahnnsa/Taa_North [Botswana]
L0d1c2a (BOT5_016) - Glui/Glui [Botswana]
L0d1c2a (TS17) - Hai//om/Hai//om [Namibia]
L0d1c (gNAB497) - Ganguela/Ganguela [Angola]
L0d1c (gNAB489) - Mumula/Nyaneka [Angola]
L0d1c1 (532) - Sekalaka/Kalanga [Botswana]
L0d1c1a2 (BOT6_278) - KwaDam/Tshwa [Botswana]
L0d1c1a2 (BOT6_121) - Naro/Naro [Botswana]
L0d1c1a2 (BOT6_286) - Shua/Shua [Botswana]
L0d1c1a2 (BOT3_03) - Hoan/Hoan [Botswana]
L0d1c1a2 (BOT2_003) - Tshaasi/Taa_east [Botswana]
L0d1c1a2 (BOT2_005) - Tshaasi/Taa_east [Botswana]
L0d1c1a2 (BOT6_007) - Tshaasi/Taa_east [Botswana]
L0d1c1a2 (BOT6_076) - Naro/Naro [Botswana]
L0d1c1a2 (BOT6_077) - Naro/Naro [Botswana]
L0d1c1a2 (BOT6_277) - KwaDam/Tshwa [Botswana]
L0d1c1a2 (BOT6_280) - KwaDam/Tshwa [Botswana]
L0d1c1a2 (XO1) - Tuu/Tuu [Namibia]
L0d1c1a2 (BOT5_008) - Glui/Glui [Botswana]
L0d1c1a2 (BOT5_038) - Glui/Glui [Botswana]
L0d1c1a2 (BOT3_06) - Hoan/Hoan [Botswana]
L0d1c1a2 (BOT6_075) - Naro/Naro [Botswana]
L0d1c1a2 (BOT2_046) - !Xoon/Taa_West [Botswana]
L0d1c1a2 (BOT6_298) - KwaDam/Tshwa [Botswana]
L0d1c1a2 (BOT6_309) - Tchwaa-Dam/Tshwa [Botswana]
L0d1c1a2 (BOT6_272) - Glui/Glui [Botswana]
L0d1c1a (NAM392) - HaillomCentral/Haillom [Namibia]
L0d1c1a1a (BOT2_053) - !Xoon/Taa_West [Botswana]
L0d1c1a1 (BOT6_161) - Mbukushu/Mbukushu [Botswana]
L0d1c1a1b (NB8) - Ju/'hoan/Ju/'hoan [Namibia]
L0d1c1a1b (NN13) - Ju/'hoan/Ju/'hoan [Namibia]
L0d1c1a1b (WB550) - Damara/Damara [Namibia]
L0d1c1a1b (NAM160) - !Xuun/!Xuun [Namibia]
L0d1c1a1b (NAM226) - HaillomCentral/Haillom [Namibia]
L0d1c1a1b (NAM209) - HaillomWest/Haillom [Namibia]
L0d1c1a1b (NAM354) - Herero/Herero [Namibia]
L0d1c1a1b (BOT6_067) - Julhoan_South/Julhoan_South [Botswana]
L0d1c1a1b (BOT6_072) - Julhoan_South/Julhoan_South [Botswana]
L0d1c1a1b (BOT6_046) - Naro/Naro [Botswana]
L0d1c1a1b (NAM216) - HaillomCentral/Haillom [Namibia]
L0d1c1a1b (NAM235) - HaillomCentral/Haillom [Namibia]
L0d1c1a1b (NAM050) - Julhoan_South/Julhoan_South [Namibia]
L0d1c1a1b (NAM053) - Julhoan_South/Julhoan_South [Namibia]
L0d1c1a1b (NAM317) - Kai-//khaun/Nama [Namibia]
L0d1c1a1b (NAM191) - !Xuun/!Xuun [Namibia]
L0d1c1a1b (BOT6_153) - Shua/Shua [Botswana]
L0d1c1a1b (BOT6_312) - Tchwaa-Dam/Tshwa [Botswana]
L0d1c1a1b (TS21) - Hai//om/Hai//om [Namibia]
L0d1c1a1b (TS24) - Hai//om/Hai//om [Namibia]
L0d1c1a1b (NAM129) - !Xuun/!Xuun [Namibia]
L0d1c1a1b (NAM125) - HaillomCentral/Haillom [Namibia]
L0d1c1a1b (NAM118) - !Xuun/!Xuun [Namibia]
L0d1c1a1a (BOT5_027) - Gllana/Gllana [Botswana]
L0d1c1a1a (BOT2_048) - !Xoon/Taa_West [Botswana]
L0d1c1a1a (BOT2_055) - !Xoon/Taa_West [Botswana]
L0d1c1a1a (BOT6_055) - !Xoon/Taa_North [Botswana]
L0d1c1a1a (NAM361) - !Xuun/!Xuun [Namibia]
L0d1c1a1a (NAM130) - !Xuun/!Xuun [Namibia]
L0d1c1a1a2 (BOT5_025) - Glui/Glui [Botswana]
L0d1c1a1a2 (BOT6_276) - KwaDam/Tshwa [Botswana]
L0d1c1a1a2 (NAM501) - !Xoon/Taa_West [Namibia]
L0d1c1a1a2 (BOT5_002) - Glui/Glui [Botswana]
L0d1c1a1a2 (BOT6_084) - Glui/Glui [Botswana]
L0d1c1a1a2 (BOT2_045) - Tuu_n0ahni/Taa_North [Botswana]
L0d1c1a1a2 (BOT6_025) - !Xoon/Taa_North [Botswana]
L0d1c1a1a2 (BOT2_011) - Taa_n0ahni/Taa_east [Botswana]
L0d1c1a1a (MD9) - !Xun/!Xun [Namibia]
L0d1c1a1a (NAM370) - HaillomNorth/Haillom [Namibia]
L0d1c1a1a (BOT6_126) - Julhoan_North/Julhoan_North [Botswana]
L0d1c1a1a (BOT6_146) - Julhoan_North/Julhoan_North [Botswana]
L0d1c1a1a (BOT5_030) - Glui/Glui [Botswana]
L0d1c1a1a (478) - Setswana/Kgalagadi [Botswana]
L0d1c1a1a (NAM159) - !Xuun/!Xuun [Namibia]
L0d1c1a1a (NAM119) - !Xuun/!Xuun [Namibia]
L0d1c1a1a (NAM188) - !Xuun/!Xuun [Namibia]
L0d1c1a1a (BOT5_026) - Gllana/Gllana [Botswana]
L0d1c1a1a (BOT6_229) - TcireTcire/TcireTcire [Botswana]
L0d1c1a1a (BOT6_219) - Shua/Shua [Botswana]
L0d1c1a1a (481) - Setswana/tswana [Botswana]
L0d1c1a1a1 (BOT3_13) - Hoan/Hoan [Botswana]
L0d1c1a1a1 (BOT5_019) - Gllana/Gllana [Botswana]
L0d1c1a1a1 (BOT6_088) - Glui/Glui [Botswana]
L0d1c1a1a1 (BOT3_01) - Hoan/Hoan [Botswana]
L0d1c1a1a1 (BOT3_05) - Hoan/Hoan [Botswana]
L0d1c1a1a1 (BOT3_09) - Hoan/Hoan [Botswana]
L0d1c1a1a1 (BOT3_11) - Hoan/Hoan [Botswana]
L0d1c1a1a1 (BOT3_12) - Hoan/Hoan [Botswana]
L0d1c1a1a1 (BOT6_063) - Naro/Naro [Botswana]
L0d1c1a1a1 (BOT6_255) - Danisi/Shua [Botswana]
L0d1c1a1a1 (BOT6_003) - Tshaasi/Taa_east [Botswana]
L0d1c1a1a1 (BOT2_057) - !Xoon/Taa_West [Botswana]
L0d1c1a1a1 (BOT6_281) - KwaDam/Tshwa [Botswana]
L0d1c1a1a1 (BOT5_033) - Gllana/Gllana [Botswana]
L0d1c1a1a1 (BOT5_004) - Glui/Glui [Botswana]
L0d1c1a1a1 (BOT2_007) - Tshaasi/Taa_east [Botswana]
L0d1c1a1a1 (BOT6_016) - Tshaasi/Taa_east [Botswana]
L0d1c1a1a1 (BOT3_07) - Hoan/Hoan [Botswana]
L0d1c1a1a1 (BOT3_10) - Hoan/Hoan [Botswana]
L0d1c1a1a1 (BOT6_105) - Naro/Naro [Botswana]
L0d1c1a1a (BOT2_056) - N«Äoha/Taa_West [Botswana]
L0d1c1a1a (522) - Sekalaka/Kalanga [Botswana]
L0d1c1a1a (BOT2_006) - Tshaasi/Taa_east [Botswana]
L0d1c1a1a (NAM313) - /Gobanin/Damara [Namibia]
L0d1c1a1a (WB532) - Nama/Nama [Namibia]
L0d1c1a1a (WB12) - Nama//Hai/Khau-an(Nama) [Namibia]
L0d1c1a1a (BOT6_040) - Naro/Naro [Botswana]
L0d1c1a1a (BOT6_041) - Naro/Naro [Botswana]
L0d1c1a1a (BOT6_002) - Tshaasi/Taa_east [Botswana]
L0d1c1a1a (BOT6_100) - Tshaasi/Taa_east [Botswana]
L0d1c1a1a (BOT2_035) - Tuu_n0ahnnsa/Taa_North [Botswana]
L0d1c1a1a (BOT2_039) - Tuu_n0ahnnsa/Taa_North [Botswana]
L0d1c1a1a (563) - Setswana/tswana [Botswana]
L0d1c1a1a (NAM065) - Julhoan_South/Julhoan_South [Namibia]
L0d1c1a1a (NAM074) - Julhoan_South/Julhoan_South [Namibia]
L0d1c1a1a (NAM304) - //O-Gain/Nama [Namibia]
L0d1c1a1a (BOT5_010) - Glui/Glui [Botswana]
L0d1c1a1a (BOT2_030) - Tuu_n0ahnnsa/Taa_West [Botswana]
L0d1c1a1a (BOT6_109) - Naro/Naro [Botswana]
L0d1c1a1a (BOT6_117) - Naro/Naro [Botswana]
L0d1c1a1a (NAM502) - !Xoon/Taa_West [Namibia]
L0d1c1a1a (BOT6_264) - Gllana/Gllana [Botswana]
H2a2a1 (rCRS) - -/- [-]

Fig. S5. Phylogenetic tree for L0d1c. Phylogeny was inferred using a total of 279 mtDNA, including all 139 mtDNA in the focus set and 140 additional L0d1c mtDNA from Barbieri *et al*. 2013. Haplogroups other than L0d1c are “collapsed”. Tips of L0d1c genomes are labeled with the format: Haplogroup (Isolate Name) - Language/Isolate Source [Country], where Isolate Name, Isolate Source, and Country are information included in the corresponding GenBank entries, and Language is the reported spoken language. Tip colours reflect data source: Red=current study, Green=Barbieri *et al*. 2013, Purple=Schuster *et al*. 2010, and Aqua=NCBI.
