# Supplementary material for: Revised Timeline and Distribution of the Earliest Diverged Human Maternal Lineages in Southern Africa
Source: PLoS One. 2015 Mar 25;10(3):e0121223. doi: 10.1371/journal.pone.0121223 (PMC4373779; doi:10.1371/journal.pone.0121223)
Supplement: S1 Table — (PDF) [file pone.0121223.s006.pdf]

Supporting Information Table S1

Revised timeline and distribution of the earliest diverged human maternal lineages in southern Africa

Eva K.F. Chan, Rae-Anne Hardie, Desiree C. Petersen, Karen Beeson, Riana M.S. Bornman, Andrew B. Smith and Vanessa M. Hayes

Table S1. Population identifiers used for study participants (n=182)

English identifier	People identifier	Language identifier	Tribes / nations / language subgroups[1]	Country of birth[2]	Total (n=182)	Complete mtDNA sequenced (n=77)
**KHOESAN POPULATIONS (n=67)**						
**San (n=26)**						
Ju/'hoan	Ju/'hoan	Ju group (Ju/'hoan)	-	Namibia (Botswana)	15	10
!Xun	!Xun	Ju group (!Xun)	-	Namibia (Angola)	10	7
Tuu-speakers	ND	Tuu group	-	Namibia (Botswana)	1	1
**Khoe (n=41)**						
Naro	Naro	Khoe group (Naro)	-	Namibia (Botswana)	10	1
Khwe	Khwe	Khoe group (Khwe)	-	Namibia	2	2
Hai//om	Hai//om	Khoe group (Nama)	-	Namibia	8	8
Nama	/Awa-khoin (non-Bantu people)	Nama (non-Bantu language)	/Hai-/Khauan	Namibia	11	6
Damara	‡Nû-khoin (Bantu people)	Nama (non-Bantu language)	Oorlams; Ao-Daman	Namibia	10	5
**NON-KHOESAN POPULATIONS (n=115)**						
**Southwestern Bantu (n=10)[3]**						
Herero	Ovaherero	Otjiherero	-	Namibia	1	1
Himba	Ovahimba	Otjihimba	Ovatjimba; Ovatau	Namibia	2	2
Owambo	Ambo	Oshiwambo	Ndonga, Kwambi	Namibia	5	5
Caprivian	-	-	-	Namibia	1	1
Angolan	-	-	-	Namibia (Angola)	1	1
**Southern Bantu (n=40)**						
Shona	Shona	chiShona	-	Zimbabwe	2	2
Pedi	Bapedi	sePedi	-	South Africa	3	2
Sotho	Basotho	seSotho	-		3	2
Tswana	Batswana	seTswana	-	South Africa (Botswana)	8	2
Tsonga	VaTsonga	Xitsonga	-	South Africa	1	1
Venda	Vhavenda	Tshivenda	-	South Africa	5	2
Xhosa	amaXhosa	isiXhosa	-	South Africa	16	4
Zulu	amaZulu	isiZulu	-	South Africa	2	2
**Baster/Coloured (n=65)**						
Baster	Baster (Rehoboth Baster)	(Afrikaans)[4]	-	Namibia[5]	42	6
Coloured	Coloured (South African Coloured)	(Afrikaans)[4]	-	South Africa	23	4

[1]Only population subgroups represented in this study have been included. [2]All study participants were recruited within the borders of Namibia or South Africa. Individuals may however have reported place of birth as Angola, Botswana or Zimbabwe. Brackets indicate contributions from a neighboring country. Place of birth is recorded in GenBank against the mitochondrial genome sequences. [3]Includes the unclassified Angolan. [4]Although Afrikaans is the predominant language spoken by the Baster's of Namibia and the Coloured of South Africa (although becoming more English within the younger generation of Coloured), Afrikaner is not used as a population identifier in this case and rather refers to European people (mainly South African and a lesser extent Namibians) of predominantly Dutch descent. [5]The Baster community were originally part of the pool of Cape admixed individuals that gave rise to the Coloured people before migrating north and establishing themselves as an independent population group in 1872 in the Namibian town of Rehoboth.
