# Supplementary material for: Revised Timeline and Distribution of the Earliest Diverged Human Maternal Lineages in Southern Africa
Source: PLoS One. 2015 Mar 25;10(3):e0121223. doi: 10.1371/journal.pone.0121223 (PMC4373779; doi:10.1371/journal.pone.0121223)
Supplement: S3 Table — (PDF) [file pone.0121223.s008.pdf]

Supporting Information Table S3

Revised timeline and distribution of the earliest diverged human maternal lineages in southern Africa

Eva K.F. Chan, Rae-Anne Hardie, Desiree C. Petersen, Karen Beeson, Riana M.S. Bornman, Andrew B. Smith and Vanessa M. Hayes

Table S3. Fourteen variants used to identify the major L0d/L0k haplogroups (n=105)

Site	Class I Variant	Major Haplogroup	N=105	Site	Class II Variant	Haplogroup*	N=105
L0d (T4232C), n=99							
1	G3438A	L0d1	47	6<br>7<br>-	T3618C<br>C4197T<br>(class II variants absent)	L0d1b<br>L0d1c<br>L0d1a	24<br>2<br>21
2<br>3<br>4	A3981G<br>C4025T<br>A4044G	L0d2	47	8<br>9;10<br>11<br>-	A5153G<br>A4038G; T4937C<br>G5147A<br>(class II variants absent)	L0d2a<br>L0d2c<br>L0d2d<br>L0d2b	36<br>4<br>6<br>1
5	G5460A	L0d3	5	-	-	-	5
L0k (G4541A and G4907C), n=6							
-	(assumed)	L0k1a	6	12;13<br>14	G207A; T8922C<br>T8222C	L0k1a1<br>L0k1a2	3<br>3

*Marker C4197T although assumed to represent L0d1c has the possibility of representing the new L0d1e/L0d1c4 lineage identified in this study. While assuming L0d1 lineages with absence of the L0d1b (T3618C) and L0d1c (C4197T) class II defining variants represent subgroup L0d1a, we note that these individuals may be carrying the new L0d1d lineage identified in this study.
