# Supplementary material for: Revised Timeline and Distribution of the Earliest Diverged Human Maternal Lineages in Southern Africa
Source: PLoS One. 2015 Mar 25;10(3):e0121223. doi: 10.1371/journal.pone.0121223 (PMC4373779; doi:10.1371/journal.pone.0121223)
Supplement: S4 Table — (PDF) [file pone.0121223.s009.pdf]

Supporting Information Table S4

Revised timeline and distribution of the earliest diverged human maternal lineages in southern Africa

Eva K.F. Chan, Rae-Anne Hardie, Desiree C. Petersen, Karen Beeson, Riana M.S. Bornman, Andrew B. Smith and Vanessa M. Hayes

Table S4. Table of estimated tMRCA for major mtDNA haplogroups calculated using a coding region-specific mutation rate of $1.26x10^{-8}$ (Mishmar *et al.* 2003).

		Coding Region			Whole Genome		
		Median	Lower 95% HPD[3]	Upper 95% HPD[3]	Median	Lower 95% HPD[3]	Upper 95% HPD[3]
**Coalescent Time[1]**	**N[2]**						
L0	134	173,117	146,384	204,724	224,857	193,882	258,463
L0d	76	113,342	89,932	138,442	144,004	118,666	173,549
L0d3	5	16,718	7,923	28,096	20,412	10,359	31,833
L0d1	38	57,641	43,964	74,657	80,302	64,782	99,248
L0d1a	6	21,807	12,272	33,485	27,954	17,748	39,615
L0d1b	18	43,730	31,990	56,994	63,880	48,823	80,645
L0d1c	12	33,961	21,773	48,143	50,552	35,456	67,345
L0d2	33	71,576	56,421	88,535	92,358	75,165	111,831
L0d2a	12	18,810	9,443	30,054	22,799	12,555	35,059
L0d2b	6	26,702	15,450	40,718	25,727	15,056	38,451
L0d2c	10	29,508	19,933	41,552	38,726	27,269	52,279
L0d2d[§]	5	24,009	10,972	39,299	25,354	12,922	39,600
L0k	13	45,601	30,033	62,363	63,065	46,144	83,305
L0k1	14	33,428	20,475	47,409	44,760	30,350	61,108
L0k1a	11	15,430	8,523	24,278	19,314	11,542	29,017
L0k1a1[♯]	8	10,302	5,277	16,581	11,914	6,569	13,330
L0k1a2[♯]	3	7,099	1,854	14,210	9,863	3,147	17,880
L0a	37	57,331	40,630	77,012	77,552	59,844	98,820
L0a1b	10	20,902	11,956	32,523	23,645	14,368	34,419
L0a2a2a	6	10,828	5,215	18,029	12,098	6,242	19,665
**Divergence Time[¶]**							
L0a'g		91,625	65,404	119,607	122,369	94,117	151,136
L0d1a'd		46,321	30,963	60,433	57,930	42,201	76,813

Estimates were calculated using the whole mitogenome (16,531 bases, excluding mutation hotpots) and the coding region (15,447 bases) of 146 mitochondrial genomes (including 7 Neanderthal genomes and the rCRS reference) with an uncorrelated lognormal relaxed clock and a constant population size model as a tree prior. [1]Time, in years, before present. [2]N is the number of individuals in the corresponding node included in the estimate. [3]95% Highest Probability Density is the interval in parameter space that contains 95% of the posterior probability. [§]The L0d2d (in *italic*) is a newly added haplogroup in PhyloTree Build 16 (19 Feb 2014). ♯L0k1a1 and L0k1a2 are new sister clades recently added to PhyloTree Build 16, but was absent in Build 15 (30 Sept 2012). [¶]L0g and L0d1d are new haplogroups identified in the current study; each were represented by a single individual.
