# Supplementary material for: Revised Timeline and Distribution of the Earliest Diverged Human Maternal Lineages in Southern Africa
Source: PLoS One. 2015 Mar 25;10(3):e0121223. doi: 10.1371/journal.pone.0121223 (PMC4373779; doi:10.1371/journal.pone.0121223)
Supplement: S5 Table — (PDF) [file pone.0121223.s010.pdf]

Supporting Information Table S5

Revised timeline and distribution of the earliest diverged human maternal lineages in southern Africa

Eva K.F. Chan, Rae-Anne Hardie, Desiree C. Petersen, Karen Beeson, Riana M.S. Bornman, Andrew B. Smith and Vanessa M. Hayes

Table S5. Table of estimated tMRCA for major haplogroups calculated using a whole genome-specific mutation rate of $1.67x10^{-8}$ (Soares *et al.* 2009).

		Coding Region			Whole Genome		
		Median	Lower 95% HPD[3]	Upper 95% HPD[3]	Median	Lower 95% HPD[3]	Upper 95% HPD[3]
**Coalescent Time [1]**	**N[2]**						
L0	134	132,769	110,640	155,736	172,250	149,306	198,872
L0d	76	86,741	68,064	105,485	110,310	90,485	133,025
L0d3	5	12,744	6,023	21,396	15,434	8,574	25,090
L0d1	38	44,183	33,776	57,313	61,369	49,597	76,079
L0d1a	6	16,669	9,021	25,159	21,295	13,750	30,323
L0d1b	18	33,376	24,159	43,651	48,654	37,295	61,981
L0d1c	12	26,093	16,883	37,328	38,674	26,757	51,476
L0d2	33	54,770	43,066	67,832	70,656	57,446	85,681
L0d2a	12	14,282	7,755	22,951	17,429	9,905	26,799
L0d2b	6	20,595	11,787	30,790	19,756	11,343	29,250
L0d2c	10	22,517	15,432	31,670	29,604	20,499	39,887
L0d2d[§]	5	18,234	8,653	30,191	19,515	9,950	30,497
L0k	13	34,996	23,542	47,995	48,407	34,402	63,670
L0k1	14	25,670	16,239	36,248	34,198	22,964	46,876
L0k1a	11	11,809	6,330	18,568	14,796	8,464	22,279
L0k1a1[#]	8	7,858	4,082	12,759	9,136	5,177	14,265
L0k1a2[#]	3	5,482	1,353	10,817	7,511	2,388	14,002
L0a	37	43,803	31,316	59,202	33,454	45,194	75,274
L0a1b	10	15,954	9,246	24,843	13,918	10,945	26,352
L0a2a2a	6	8,354	3,989	13,898	14,038	4,736	15,300
**Divergence Time[¶]**							
L0a'g		70,083	49,816	91,030	93,814	71,873	115,683
L0d1b'd^		37,607	28,296	48,792	-	-	-
L0d1a'd^		-	-	-	44,084	31,063	58,114

Estimates were calculated using the whole mtDNA (16,531 bases, excluding mutation hotpots) and the coding region (15,447 bases) of 146 mitochondrial genomes (including 7 Neanderthal genomes and the rCRS reference) with an uncorrelated lognormal relaxed clock and a constant population size model as a tree prior. [1]Time, in years, before present. [2]N is the number of individuals in the corresponding node included in the estimate. [3]95% Highest Probability Density is the interval in parameter space that contains 95% of the posterior probability. [§]The L0d2d (in *italic*) is a newly added haplogroup in PhyloTree Build 16 (19 Feb 2014). ♯L0k1a1 and L0k1a2 are new sister clades recently added to PhyloTree Build 16, but was absent in Build 15 (30 Sept 2012). [¶]L0g and L0d1d are new haplogroup identified in the current studyl each were represented by a single individual. [^]Missing entries (-) are due to alternate branching inferred from using whole genome vs. coding region data.
